# Catalytic Performance of Cobalt(II) Polyethylene Catalysts with Sterically Hindered Dibenzopyranyl Substituents Studied by Experimental and *MLR* Methods

**DOI:** 10.3390/molecules27175455

**Published:** 2022-08-25

**Authors:** Arfa Abrar Malik, Md Mostakim Meraz, Wenhong Yang, Qiuyue Zhang, Desalegn Demise Sage, Wen-Hua Sun

**Affiliations:** 1 CAS Key Laboratory of Engineering Plastics and Beijing National Laboratory for Molecular Science, Institute of Chemistry, Chinese Academy of Sciences, Beijing 100190, China; 2 CAS Research/Education Center for Excellence in Molecular Sciences and International School, University of Chinese Academy of Sciences, Beijing 100049, China; 3 Advanced Materials Research Center, Petrochemical Research Institute, PetroChina Company Limited, Beijing 102206, China

**Keywords:** dibenzopyranol groups, cobalt complexes, catalytic activity, *β*-H elimination, linear polyethylene, ethylene polymerization, multiple linear regression (*MLR*) analysis

## Abstract

Given the great importance of cobalt catalysts supported by benchmark bis(imino)pyridine in the (oligo)polymerization, a series of dibenzopyran-incorporated symmetrical 2,6-bis(imino) pyridyl cobalt complexes (**Co1**–**Co5**) are designed and prepared using a one-pot template approach. The structures of the resulting complexes are well characterized by a number of techniques. After activation with either methylaluminoxane (MAO) or modified MAO (MMAO), the complexes **Co1**–**Co4** are highly active for ethylene polymerization with a maximum activity of up to 7.36 × 10^6^ g (PE) mol^−1^ (Co) h^−1^ and produced highly linear polyethylene with narrow molecular weight distributions, while **Co5** is completely inactive under the standard conditions. Particularly, complex **Co3** affords polyethylene with high molecular weights of 85.02 and 79.85 kg mol^−1^ in the presence of MAO and MMAO, respectively. The ^1^H and ^13^C NMR spectroscopy revealed the existence of vinyl end groups in the resulting polyethylene, highlighting the predominant involvement of the *β*-H elimination reaction in the chain-termination process. To investigate the mechanism underlying the variation of catalytic activities as a function of substituents, multiple linear regression (*MLR*) analysis was performed, showing the key role of open cone angle (*θ*) and effective net charge (*Q*) on catalytic activity.

## 1. Introduction

Concerted efforts have been devoted in last two decades to exploring the propensity of bis(imino)pyridine metal complexes’ precatalysts towards ethylene (oligo)polymerization. This effort was driven by their distinct advantages of low cost, ease of preparation, and high polymerization activity [1,2,3,4,5]. Indeed, it is a continuous topic of interest to enhance the scope of these precatalysts in terms of polymerization activity, thermal stability, and the molecular weight of the resulting polyethylene, either through adjusting the steric and electronic substitution pattern of the benchmark bis(imino)pyridine framework or designing a completely new ligand framework [6,7,8,9,10,11,12,13,14,15]. Tremendous studies have been performed by adjusting the imine-N and imine-C substituents, as well as the focal pyridine unit, to examine their influence on the catalytic activity for ethylene polymerization [16,17,18,19,20,21,22,23,24,25,26,27,28]. In particular, more focus was placed on the incorporation of the bulky substituents at the axial sites of the chelate ring in order to improve the chain propagation versus the chain transfer reactions at elevated reaction temperatures [17,19,28].

The benzhydryl substituent is one of the substituents of the bis(imino)pyridine cobalt complexes that have been investigated to explore the extent of the complex catalyst space. This class of catalyst system experienced difficulties in depicting high catalytic activity and thermal stability simultaneously. However, these cobalt catalysts can produce characteristically linear and high-density polyethylene with a high molecular weight and broad to narrow distributions [9,10,11,12]. Furthermore, different substitutions at the *para*-position of *N*-aryl rings of cobalt complex systems yield different products ranging from oligomers to polymers. To this end, many works regarding the modification of substitutions at the *para*-position of *N*-aryl rings in bis(imino)pyridyl cobalt complexes have been reported [29,30,31,32,33]. These modified cobalt complexes result in a drastic change of catalytic activity and molecular weight of the produced product, attributed to the steric and electronic effect of the induced species. For example, Wu and coworkers studied the influence of electronic and steric effects at the *para*-position [7]. Their results exhibited no significant change in activity on the basis of electronic perturbations. Equally, variation in the steric substituents makes a notable contribution to enhancing the molecular weights of the resulting polymer. Likewise, the Li group noticed a large difference in catalytic performance with variations in the steric hindrance at the *para*-position of the *N*-aryl units (H < Br < iPr) [32]. It is worth mentioning that the precatalysts with a sterically enhanced bulky group such as electron-donating (iPr) and electron-withdrawing (Br) substituents at the *para*-position of the imines usually display superior activities and molecular weights compared to precatalysts with a hydrogen substituent.

**Chart 1:**
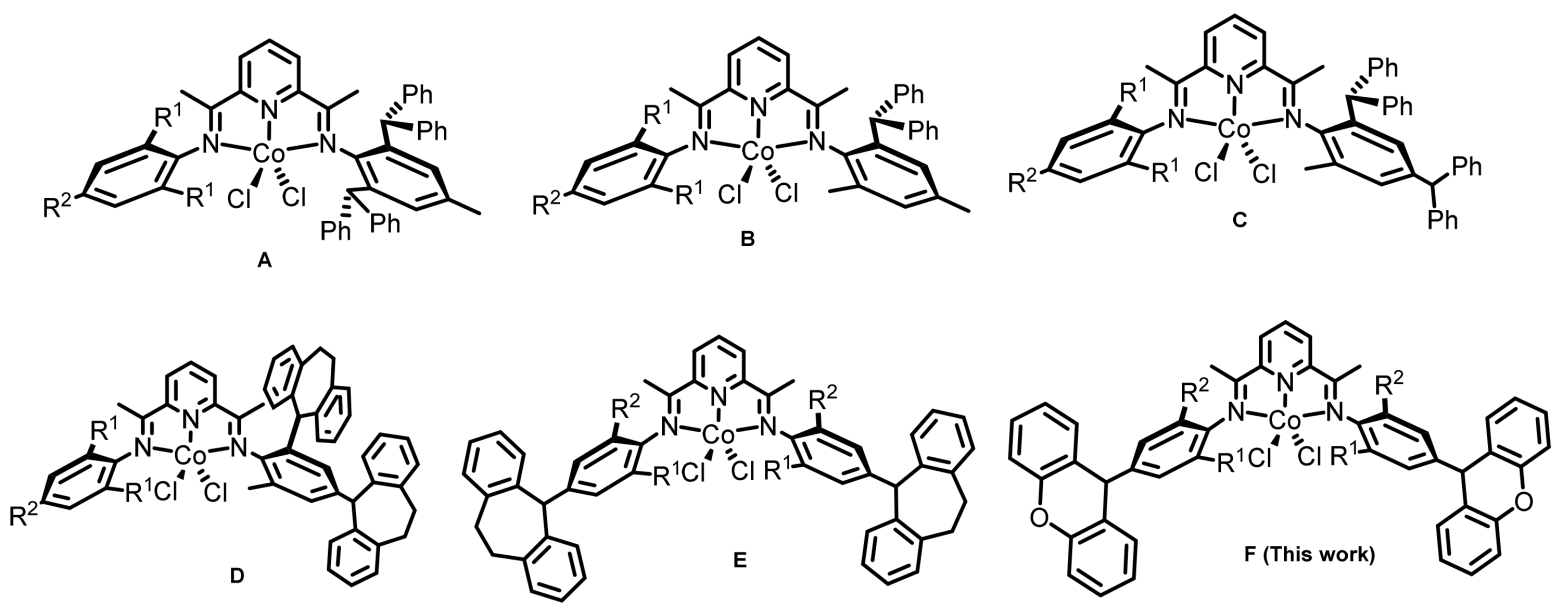
Some examples of previously reported bis(imino)pyridine cobalt chloride precatalysts, (**A**–**E**), along with current work (**F**).

With this development, our group studied the influence of different steric groups on the bis(imino)pyridine metal precatalysts framework. Firstly, catalysts with an *ortho*-benzhydryl substituted group as shown in Chart 1A [9] exhibited high polymerization activity up to 9.87 × 10^6^ g (PE) mol^−1^ (Co) h^−1^, and the molecular weight of resulting polyethylene fall in the range of 100–330 kg mol^−1^ with narrow molecular weight distributions. However, the incorporation of a benzhydryl substituent at only one *ortho*-position provided a slightly lower activity of 2.85 × 10^6^ g (PE) mol^−1^ (Co) h^−1^, in addition to producing a lower-molecular-weight polyethylene in the range of 53.81–57.56 kg mol^−1^ (Chart 1B) [11]. Moreover, introducing a benzhydryl substituent at the *para*-position within the same *N*-aryl group results in high activity (1.81 × 10^7^ g (PE) mol^−1^ (Co) h^−1^) and molecular weight (118 kg mol^−1^) of the resultant polyethylene exhibiting the unimodal behavior (Chart 1C) [12]. Similarly, the successful incorporation of dibenzocycloheptyl groups at the *ortho*- and *para*-positions of *N*-aryl rings attached to the imine nitrogen atom in the generic bis(imino)pyridine yielded polyethylene with high activity up to 1.00 × 10^7^ g (PE) mol^−1^ (Co) h^−1^ and thermally stability at 60 °C. This report depicted the high thermal stability for unsymmetrical 2,6-bis(imino)pyridylcobalt (II) chloride precatalysts (Chart 1D) [29]. The activity of **D** is lower than that of **C**, because of the steric hindrance caused by the bulky dibenzocycloheptyl group, which inhibits the incoming monomer to approach the central metal atom. In addition to that, our research group reported a series of symmetrical cobalt complexes with a dibenzocycloheptyl substituent at the *para* position of the *N*-aryl rings. These cobalt complexes yielded highly linear polyethylene with catalytic activity up to 1.21 × 10^7^ g (PE) mol^−1^ (Co) h^−1^ and narrow molecular distribution (Chart 1E) [33].

With a view to further enhance the polymerization scope of these cobalt precatalysts toward ethylene polymerization, we were interested in functionalizing the bis(imino)pyridine with dibenzopyran bulky group as the *para*-substituent on the *N*-aryl group (Chart 1F). For this purpose, a series of symmetrical 2,6-bis(imino) pyridyl cobalt complexes with a *para*-substituted dibenzopyran group are prepared. The synthesized cobalt complexes are fully characterized, and extensive ethylene polymerization is performed for investigating the impact of structural changes on catalytic performance. The effect of a co-catalyst, temperature, and pressure on the catalytic activity is examined during the polymerization runs. In addition, the properties of polymers, including molecular weight, polydispersity, melting temperature, and microstructure, are well described. To gain a better understanding of the variation mechanism regarding the catalytic activity as a function of substituents, multiple linear regression (*MLR*) analysis is carried out to unravel the role of electronic and steric effects, thus providing a guide for the future design of novel catalysts with enhanced catalytic activity.

## 2. Results

### 2.1. Synthesis and Characterization of ***Co1**–**Co5***

**Scheme 1:**
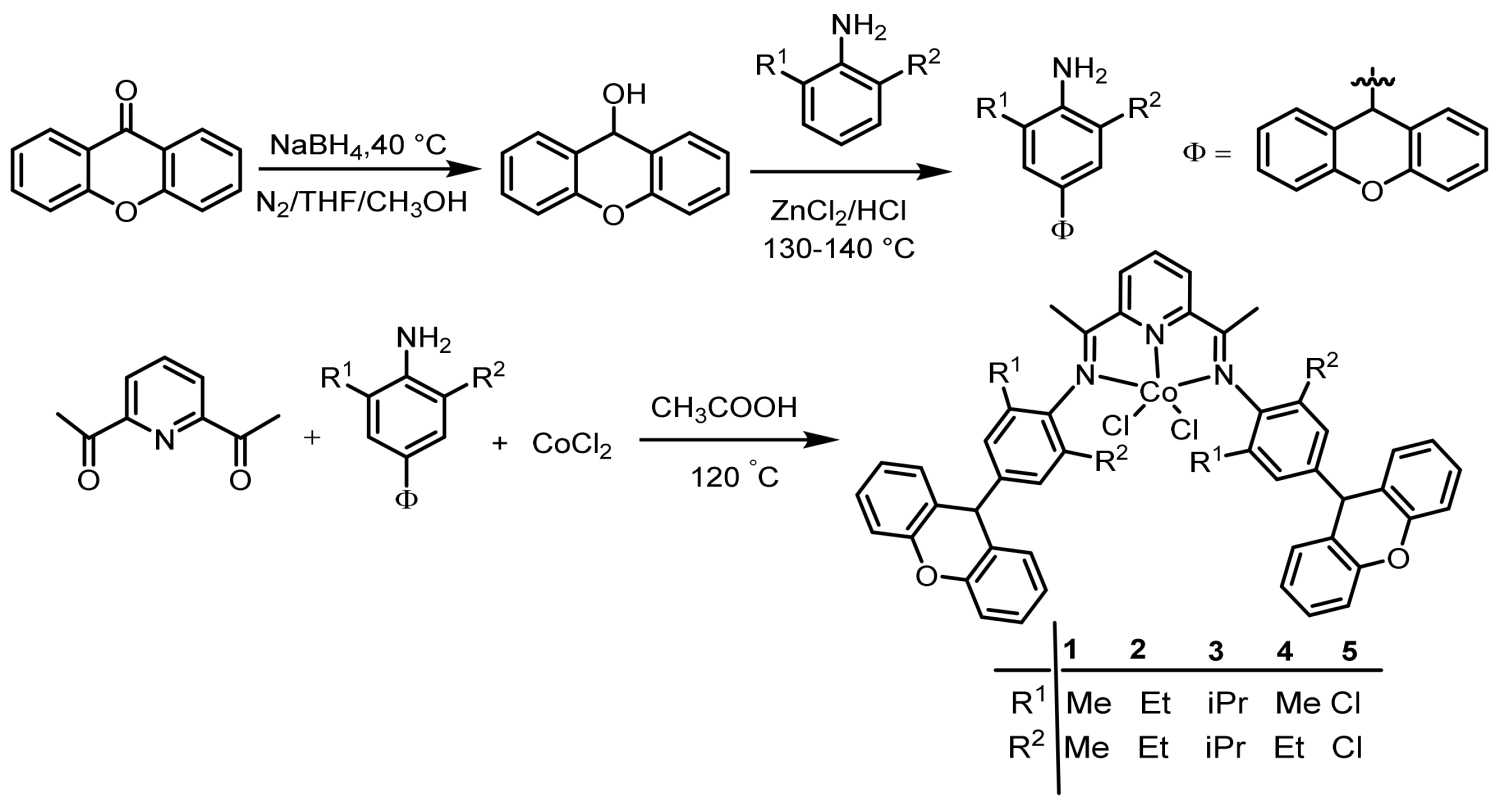
Synthesis of bulky anilines and the corresponding cobalt(II) chloride complexes (**Co1–Co5**).

**Figure 1:**
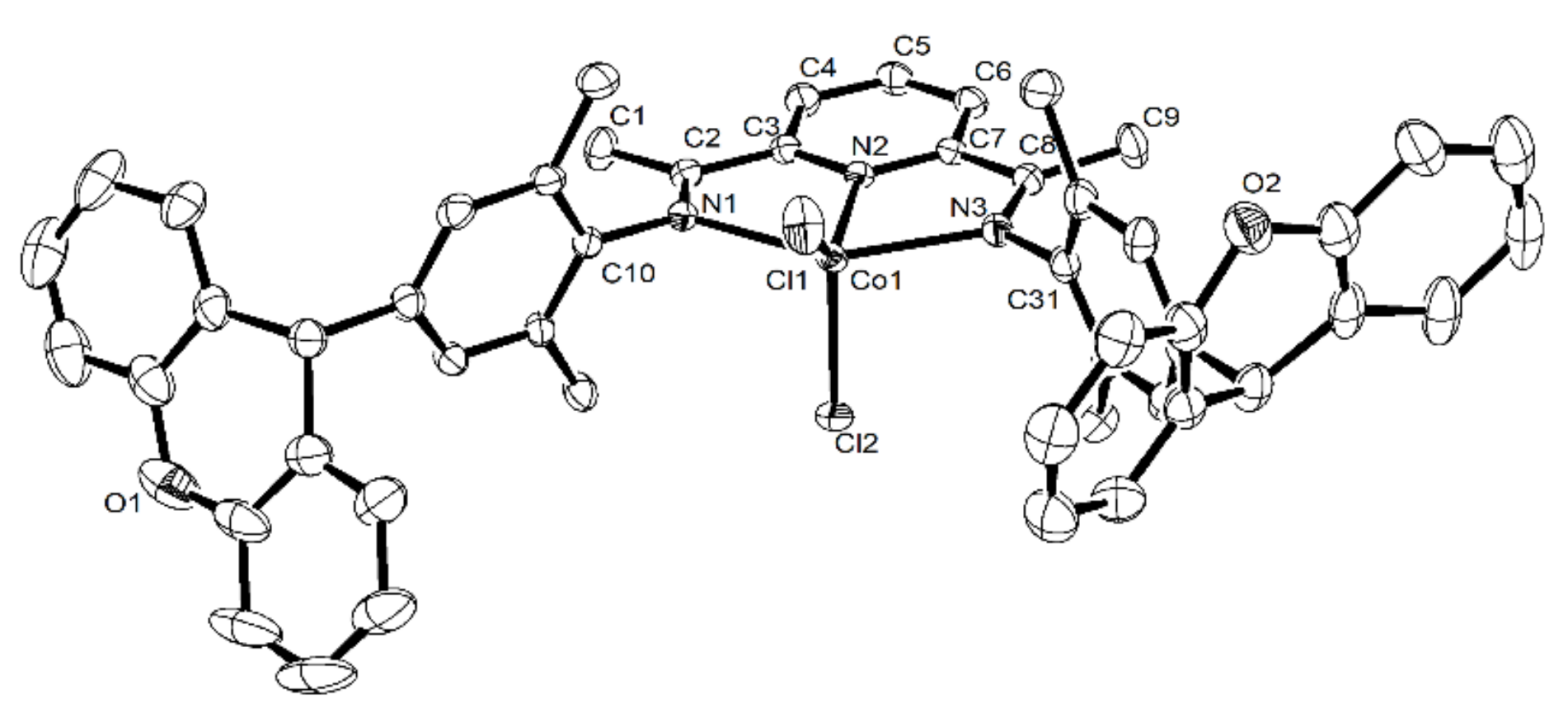
Molecular structure of **Co1** with thermal ellipsoids adjusted at the 30% probability level, and hydrogen atoms are omitted for clarity.

**Figure 2:**
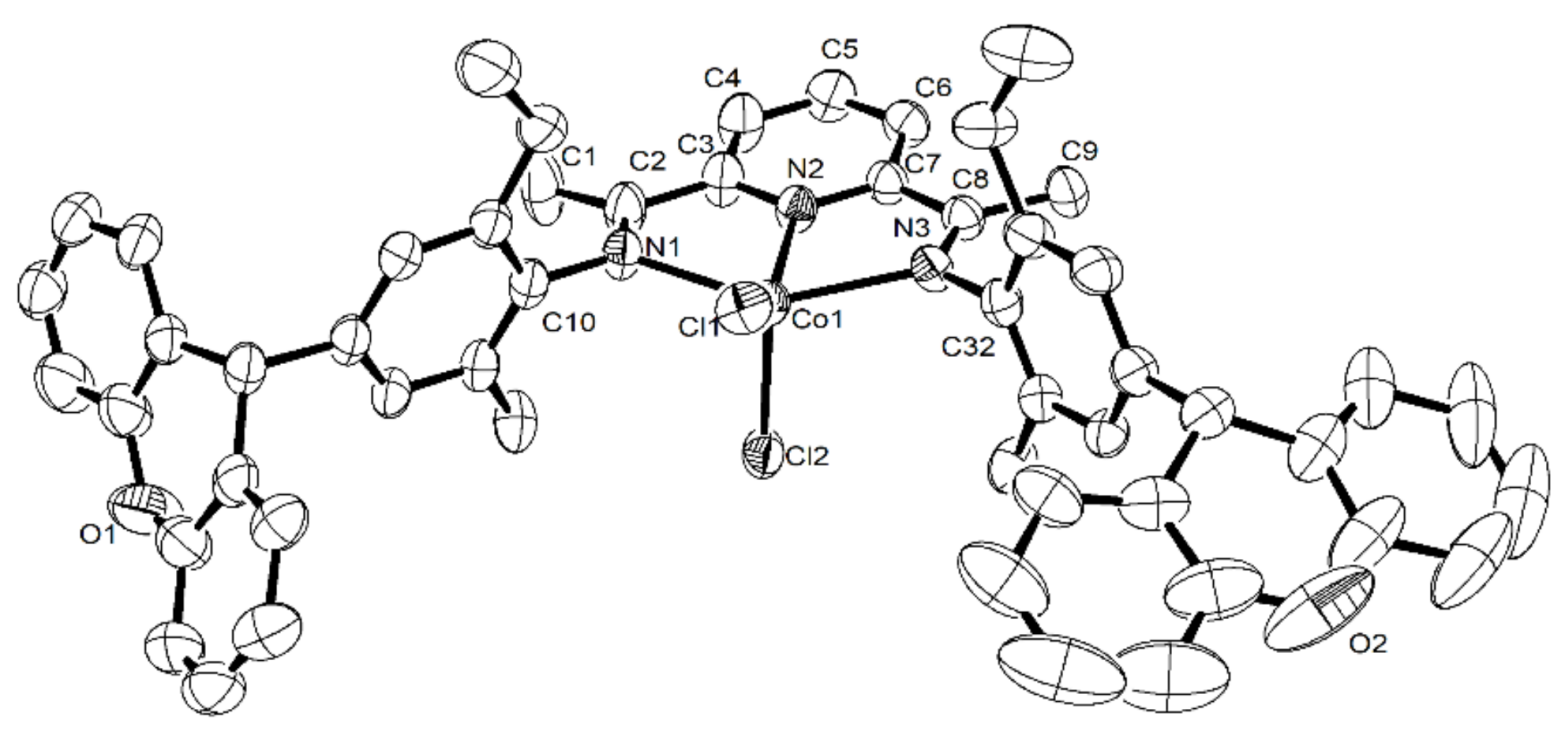
Molecular structure of **Co4** with thermal ellipsoids adjusted at the 30% of probability level, and hydrogen atoms are omitted for clarity.

As shown in Scheme 1, various 4-dibenzopyranol-2,6-dialkyllanilines were prepared from treating corresponding dialkylanilines with dibenzopyranol. The title cobalt complexes (**Co1**–**Co5**) were obtained in the one-pot reactions of 2,6-diacetylpyridine, 4-dibenzopyranol-2,6-dialkyllanilines, and CoCl_2_ refluxed with acetic acid. The cobalt complexes were precipitated with an excess of diethyl ether and isolated as an air-stable green powder in high yields. The representative complexes **Co1** and **Co4** were the subject of single-crystal X-ray diffraction analysis. The FT-IR spectra of complexes (**Co1**–**Co5**) showed signals located in the region from 1602 to 1652 cm^−1^ ascribed to the stretching vibration of C=N_imine_ bonds. This information demonstrated the active coordination between the central metal and the donor atoms. The elemental analysis of all the complexes exhibited their consistency with the molecular formula. Moreover, the molecular structures of **Co1** and **Co4** were well described using single-crystal X-ray diffraction analysis as shown in Figure 1 and Figure 2.

Single crystals of **Co1** and **Co4** subjected to X-ray determination were obtained by dissolving them slowly in *n*-hexane, which was further treated with the solution of the corresponding complex in dichloromethane. The crystal structures of **Co1** and **Co4** are shown in Figure 1 and Figure 2, and the selected values of bond length and bond angles are listed in Table 1. The structures of **Co1** and **Co4** are fundamentally similar, because both have a five-coordinate structure system with the cobalt center surrounded by two chloride atoms and three nitrogen atoms from the bis(arylimino)pyridine. In both cases, a tridentate ligand with imino and pyridine moieties is basically planar; the *N*-aryl groups are inclined almost perpendicularly to the bis(imino)pyridine plane. The geometry can be explained well by the tau value (τ_5_) calculated by the equation (*β* − *α*)/60, where *β* is the largest, and *α* is the second-largest angle in the coordination sphere [29]. According to the τ_5_ values (0.21 for **Co1** and 0.07 for **Co4**), the crystal structures of the complexes described the bonding of a single cobalt center with bis(arylimino)pyridine ligands and two chlorine atoms leading to distorted square-pyramidal and perfect square-pyramidal geometry, respectively.

The basal plan includes all the nitrogen atoms (N1, N2, and N3) and one chlorine atom Cl(1), while the second chlorine atom Cl(2) forms the apical position. The dihedral angles of **Co1** and **Co4** are 82.55° and 77.87°, respectively, where the cobalt atoms exist above the N1, N2, and N3 planes. These results can be found in previously reported studies [29,33,34,35]. Different substitutions of aryl rings connected with imine nitrogen atoms on both sides cause the change in steric hindrance around their atoms leading to a slight change in bond lengths and bond angles. The bond lengths for Co–N_imine_ atoms are 2.223(3) Å and 2.225(3) Å in the **Co1** complex, while these bond length values are a little bit higher in **Co4** complex, i.e., 2.239(4) Å and 2.246(4) Å. The fluctuation of bond lengths in each complex is due to the presence of different substitutions on *N*-aryl rings. Moreover, further description reveals that the Co–N_pyridine_ bond is comparatively stronger than the Co–N_imine_ bonds for **Co1** and **Co4** complex systems. The length N(3)–C(8) bonds in both complexes describe C=N.

### 2.2. Ethylene Polymerization

The microstructure of the resultant polyethylene may sometimes vary due to the variation of co-catalysts. Primarily, different alkylaluminium co-catalysts were tried to check their catalytic activity, and the results indicated that methylaluminoxane (MAO) or modified methylaluminoxane (MMAO) are more effective co-catalysts than other alkyl aluminium reagents [36,37]. **Co1** was chosen as a precatalyst for the initial screening and finding of the standard conditions in order to examine the catalytic performance of the cobalt precatalysts toward ethylene polymerization. Different reaction conditions such as temperature, Al/Co ratio, and time were steadily varied under an ethylene pressure of 10 atm using methylaluminoxane (MAO) or modified methylaluminoxane (MMAO) as cocatalysts. The set of optimal reaction conditions was extended to predict the catalytic performance of the rest of the cobalt precatalysts **Co2**–**Co5**. Furthermore, **Co1** was also screened at an ethylene pressure of 5 and 1 atm. Polymer characterization was carried out using different techniques. The molecular weight (*M*_w_) and molecular weight distribution (*M*_w_/*M*_n_) of the resultant polyethylene were determined using gel permeation chromatography, and the melting temperature was measured using differential scanning calorimetry (DSC). The microstructure analysis of resultant polyethylene samples was performed using high-temperature NMR (^1^H/^13^C) spectroscopy.

#### 2.2.1. Catalytic Evaluation Using **Co1** with MAO as a Cocatalyst

**Figure 3:**
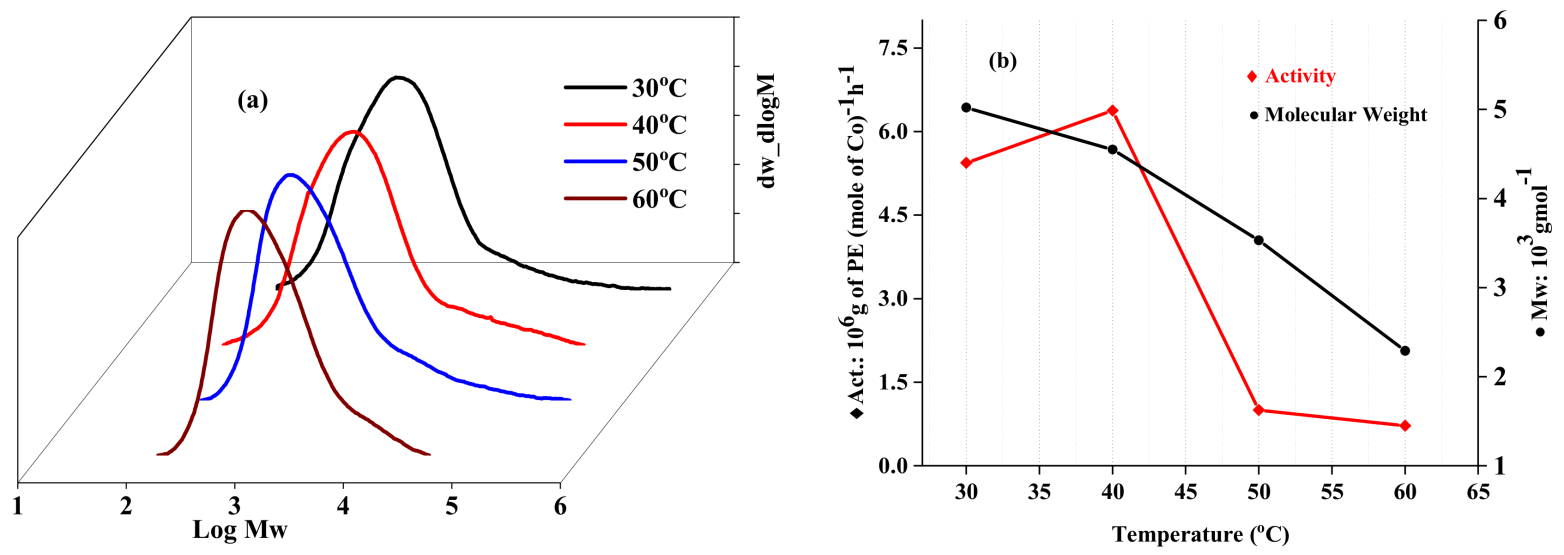
GPC curves of the obtained polyethylene (**a**); activity and *M*_w_ as a function of reaction temperature (**b**) for the **Co1**/MAO system (Table 2, entries 1–5).

Ethylene polymerization was carried out by optimizing **Co1** in toluene under the ethylene pressure of 10 atm, and all the results are listed in Table 2. Considering different conditions, the first polymerization run was performed at different temperatures ranging from 30 to 70 °C. During all the running at different temperatures, the Al/Co ratio was constant with the reaction time of 30 min (Table 2, entries 1–5, runs 1). By increasing the temperature from 30 °C onwards, the highest activity was observed at 40 °C and then suddenly started to decrease until there was no activity at 70 °C. This behavior exhibits the sensitivity of catalytic activity towards high temperatures. This may be attributed to the low solubility of ethylene at high temperatures and the deactivation ion of cobalt’s active species. On the contrary, the molecular weight of produced polyethylene decreases from 5.02 kg mol^−1^ to 2.29 kg mol^−1^ with an increase in temperature. This decline may happen due to a high chain-transfer rate and low ethylene concentration at elevated temperatures [36]. Moreover, Figure 3a depicts the unimodal behavior of GPC curves at different temperatures, and Figure 3b shows the clear graphical variation of activity and molecular weight with temperatures corresponding to entries 1–5 of Table 2.

**Figure 4:**
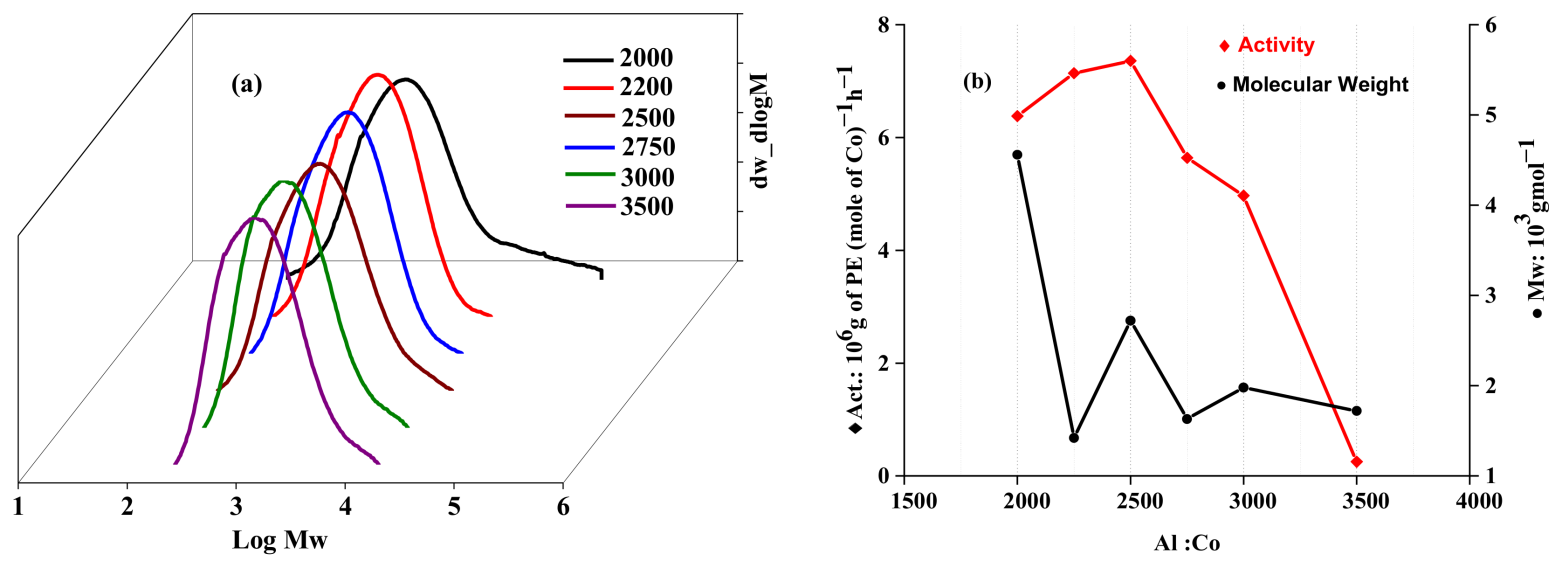
GPC curves of the obtained polyethylene (**a**); activity and *M*_w_ as a function of Al/Co ratio (**b**) for the **Co1**/MAO system (Table 2, entries 2, 6–10).

Furthermore, the variations of the molar ratio of Al/Co were tried from 2000 to 3500 at 40 °C and 30 min (Table 2, entries 2, 6–10). The highest value of the activity (7.36 × 10^6^ g (PE) mol^−1^ (Co) h^−1^) was observed at an Al/Co of 2500. Additionally, the polymerization activity was decreased by increasing the concentration of MAO. The molecular weight of the resultant PE ranges from 1.42–2.27 kg mol^−1^, as shown in Figure 4.

**Figure 5:**
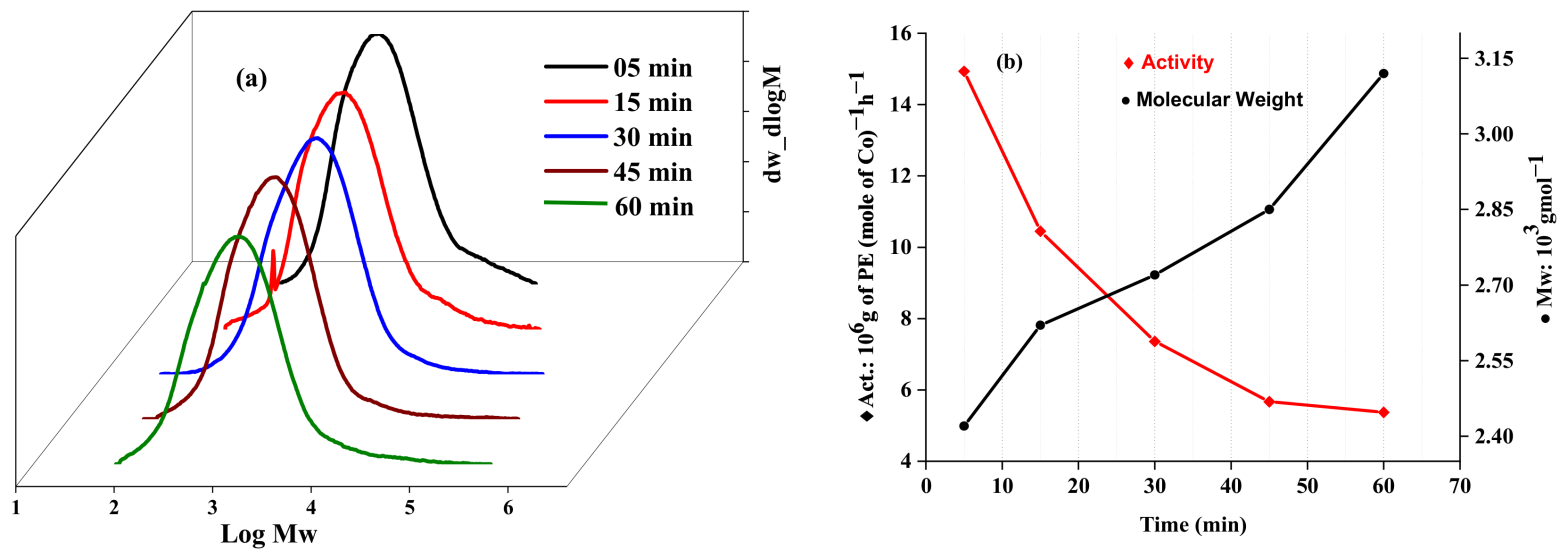
GPC curves of the obtained polyethylene (**a**); activity and *M*_w_ as a function of reaction time (**b**) for the **Co1**/MAO system (Table 2, entries 7, 11–14).

The effect of reaction time from 5 to 60 min on the polymerization process was examined at optimum conditions, i.e., temperature at 40 °C and Al/Co ratio of 2500 (Table 2, entries 7, 11–14). The slow decrease of activity from 14.93 × 10^6^ g (PE) mol^−1^ (Co) h^−1^ (5 min) to 5.37 × 10^6^ g (PE) mol^−1^ (Co) h^−1^ (60 min) is due to some deactivation of the active species over longer time spans of reactions. It is unlikely that the molecular weight of PE showed a slight change with time, and it ranged from 2.42–3.12 kg mol^−1^; this may happen due to a chain transfer and the *β*-H elimination processes. The slight increase in molecular weight of polyethylene with reaction times is plotted in the form of GPC curves as shown in Figure 5a. The unimodal molecular weight distribution was observed for a resultant product with a melting point ranging from 121.9–124.8 °C. The plots of activity and the molecular weight of polyethylene as a function of the reaction time are given in Figure 5b. These dependencies exhibit similarities with related catalytic systems [10,11,12,13].

To investigate the effect of ethylene pressure on the catalytic activity and molecular weight of the resultant polyethylene, another polymerization run was performed (Table 2, entries 15 and 16) at optimized reaction conditions (T = 40 °C, Al/Co = 2500, t = 30 min) at 1 and 5 atm, respectively. There is a strong influence of pressure on both activity and molecular weight as shown in Figure S1 of Supplementary Materials. The GPC trace exhibits the unimodal curves of the resultant polymer with the melting point of 124.8 °C and 121.9 °C at 1 and 5 atm, respectively.

#### 2.2.2. Catalytic Evaluation Using **Co1** with MMAO as a Cocatalyst

**Figure 6:**
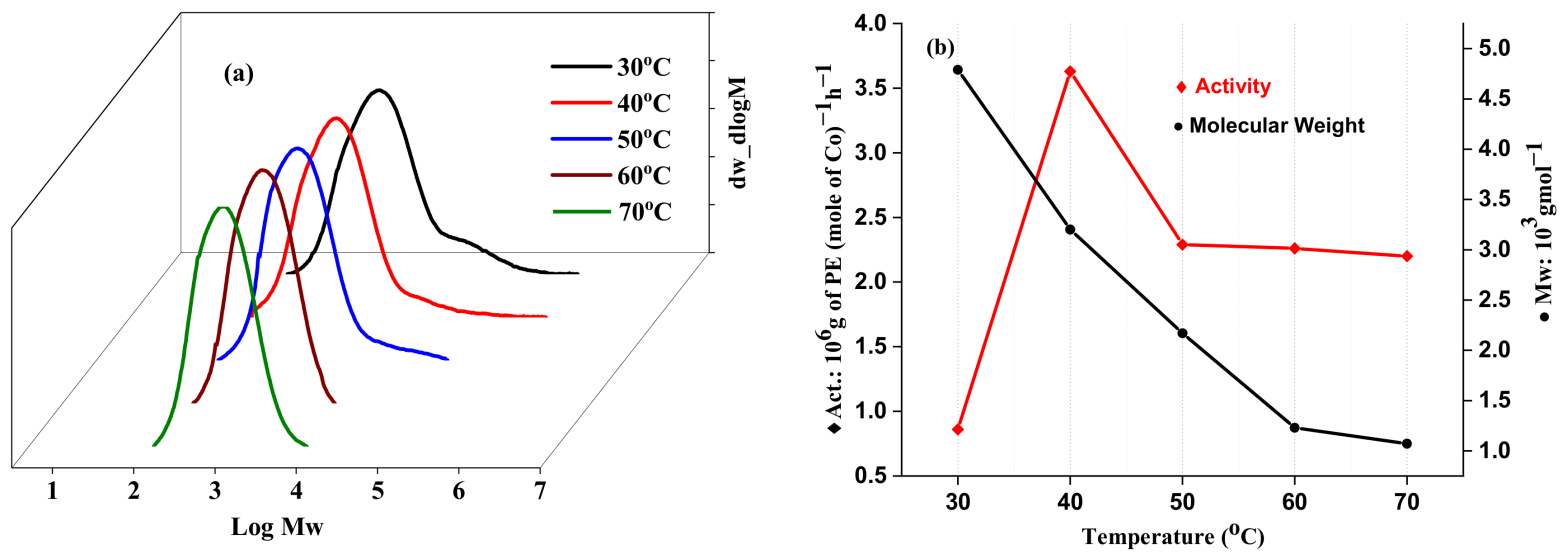
GPC curves of the obtained polyethylene (**a**); activity and *M*_w_ as a function of reaction temperature (**b**) for the **Co1**/MMAO system (Table 3, entries 1–5).

The ethylene polymerization process for **Co1**/MMAO was conducted in the same manner as the **Co**/MAO system. Following the same procedure, the molar ratio of Al/Co was fixed at 2000, and the ethylene polymerization was performed in toluene by adjusting the temperature from 30 °C to 70 °C under 10 atm pressure and over 30 min reaction time (Table 3, entries 1–5). The highest value of activity is achieved at 40 °C. Furthermore, there is a gradual decrease in activity and molecular weight with increasing temperature, and this trend corresponds to the results obtained by the MAO cocatalyst system (Figure 6).

**Figure 7:**
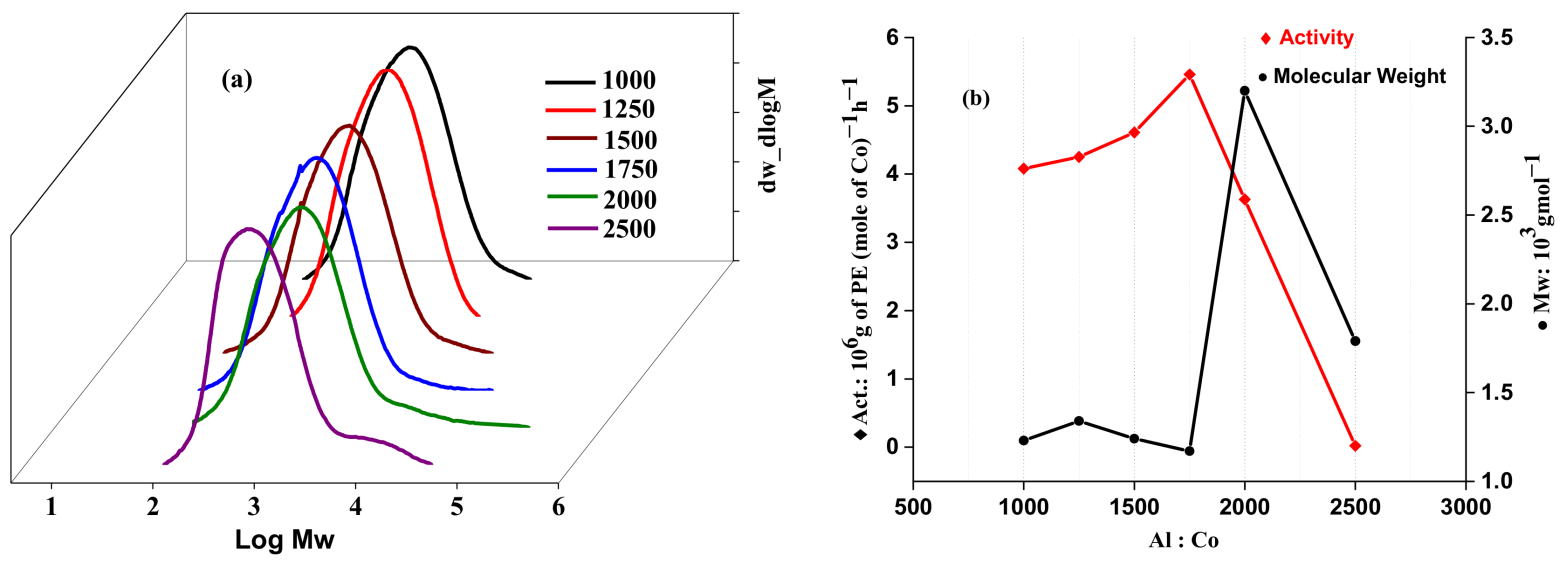
GPC curves of the obtained polyethylene (**a**); activity and *M*_w_ as a function of Al/Co ratio (**b**) for the **Co1**/MMAO system (Table 3, entries 6–10).

By fixing the optimum temperature at 40 °C, the second series of ethylene polymerization was performed by changing the Al/Co ratio from 1000 to 2500 (Table 3, entries 2, 6–10). The polymerization activities varied from 0.02–5.46 × 10^6^ g (PE) mol^−1^ (Co) h^−1^ with the highest active peak at the molar ratio of 1750. There is no regularity of the variation of molecular weight as depicted by GPC traces, as shown in Figure 7, with the PDI value of resultant PEs around 2.0.

**Figure 8:**
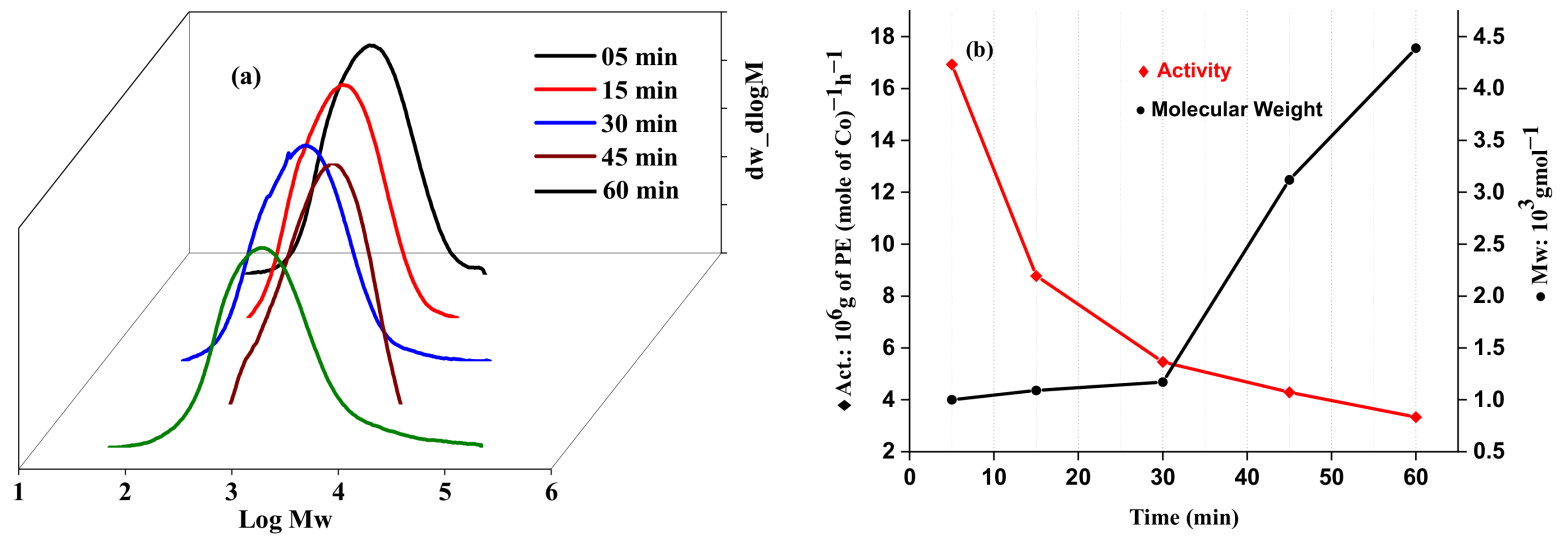
GPC curves of the obtained polyethylene (**a**); activity and *M*_w_ as a function of reaction time (**b**) for the **Co1**/MMAO system (Table 3, entries 11–14).

The reaction time was prolonged from 5 to 60 min in the third series of ethylene polymerization (Table 3, entries 11–14, run 3). By increasing the reaction time, the catalytic activity decreased from 16.93 to 3.33 × 10^6^ g (PE) mol^−1^ (Co) h^−1^, which could possibly be due to the deactivation of active species. Figure 8 shows the GPC trace of the resultant PEs, and, clearly, there is an inverse relationship between molecular weight and activity as a function of reaction time.

There is a significant effect of ethylene pressure on the catalytic activity and molecular weight of the produced polyethylene as shown in Figure S2 in the Supplementary Materials, exhibiting the similarity of the trend in comparison to those observed for the **Co1**/MAO system.

#### 2.2.3. Catalytic Evaluation Using **Co1**–**Co5** with MAO and MMAO as the Co-Catalysts

**Figure 9:**
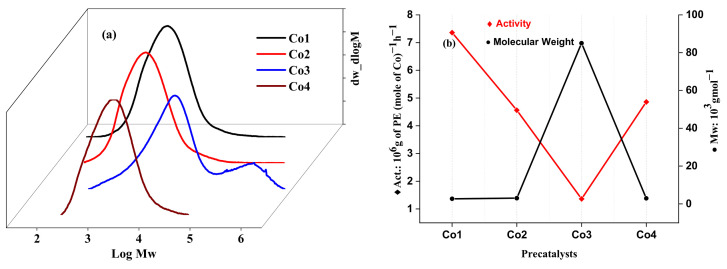
GPC curves of the obtained polyethylene (**a**); activity and *M*_w_ for different precatalysts at the optimized reaction conditions (**b**) for the **Co1**–**Co4**/MAO system (Table 4, entries 1–4).

After optimizing the reaction conditions based on **Co1**/MAO system, the behavior of the remaining cobalt precatalysts **Co2**–**Co5** toward ethylene polymerization was investigated under optimized reaction conditions, i.e., the reaction temperature of 40 °C, the Al/Co ratio of 2500, ethylene pressure of 10 atm, and the run time of 30 min as listed in entries 2–5 of Table 4. All the complexes exhibit good activity with values ranging from 1.36 to 7.36 × 10^6^ g(PE) mol^−1^ (Co) h^−1^ towards the ethylene polymerization, except for the **Co5** complex, which is probably due to the different electronic negativity of the *ortho*-substituents within the *N*-aryl group. The steric effect has a significant influence on catalytic activities. The hindrance of *ortho*-substituent due to bulky groups decreases the activity of the resultant polymer in the following order **Co1** (Me, Me) > **Co4** (Me, Et) > **Co2** (Et, Et) > **Co3** (iPr, iPr). Thus, the highest value of activity 7.36 × 10^6^ g(PE) mol^−1^ (Co) h^−1^ obtained by the **Co1** (Me, Me) precatalyst bearing the least bulky substituents. However, **Co3** (i-Pr, i-Pr) displayed the least value of activity 1.36 × 10^6^ g(PE) mol^−1^ (Co) h^−1^. The *iso*-propyl substituent at the *ortho*-positions of *N*-aryl rings hinders the incoming ethylene monomers and affects the coordination, leading to a decrease of activity. Meanwhile, the substituent in the *ortho*-position of *N*-aryl group strongly influences the molecular weight of the resulting polyethylene. The highest value of molecular weight 85.02 kg mol^−1^ for **Co3** corresponds to the bulkiest *iso*-propyl *N*-aryl group. This shows that bulky substituents protect the active sites and suppress the chain transfer. Similar behavior was observed in previous reports [9,10,11,12,33]. Moreover, the melting point of the polyethylene produced by the **Co3** precatalyst is 134.9 °C, which is the highest value among all cobalt complexes. This could be attributed to the polymer’s high crystallinity. Figure 9a shows that all the PEs obtained from **Co1**, **Co2**, and **Co4** possessed narrow distributions (PDI ≈ 2.0), and their GPC curves show unimodal distribution possessing a single-site catalyst system. The GPC trace of PE from **Co3** demonstrated the bimodal distribution, which is due to the multisite active species during the polymerization process. The values of the activities and molecular weight of different cobalt precatalysts at the optimized reaction conditions are given in Figure 9b.

**Figure 10:**
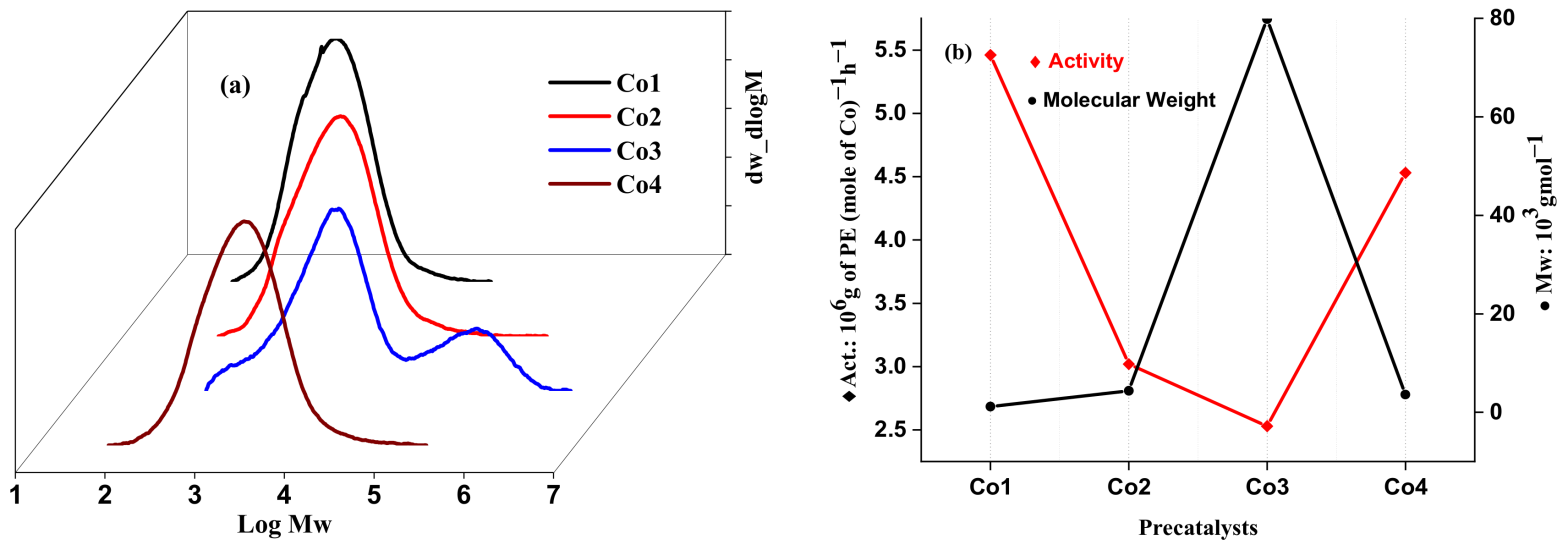
GPC curves of the obtained polyethylene (**a**); activity and *M*_w_ for different precatalysts at the optimized reaction conditions (**b**) for the **Co1**–**Co4**/MMAO system (Table 4, entries 6–9).

For the MMAO cocatalyst, ethylene polymerization was conducted at the optimized conditions, i.e., reaction temperature of 40 °C, Al/Co ratio of 1750, ethylene pressure of 10 atm, and reaction time of 30 min for the remaining cobalt complexes with the decreasing order of activity **Co1** (Me, Me) > **Co4** (Me, Et) > **Co2** (Et, Et) > **Co3** (i-Pr, i-Pr). All the cobalt complexes bearing polymers are unimodal, describing the single-site active species, except **Co3** as shown in Figure 10a. The trend of activity and the molecular weight Co/MMAO system are similar to Co/MAO. **Co3** produces polyethylene with a very high molecular weight of 79.85 kg mol^−1^; the molecular weights of the remaining cobalt complexes range from 1.17 to 4.38 kg mol^−1^. This high value for **Co3** depicts that steric hindrance of the *ortho*-substituent of the *N*-aryl group dominatingly affect the molecular weight of polymer describing the bulky groups are responsible for suppressing the chain transfer. Additionally, the **Co3** precatalyst exhibits the lowest catalytic activity as listed in Table 4 and displays a broader and bimodal molecular weight distribution corresponding to Figure 10. Comparatively, MAO generates polyethylene of a much higher molecular weight than the system containing MMAO, shown as entries 3 and 8 of Table 4.

### 2.3. Microstructural Properties of Polyethylene

**Figure 11:**
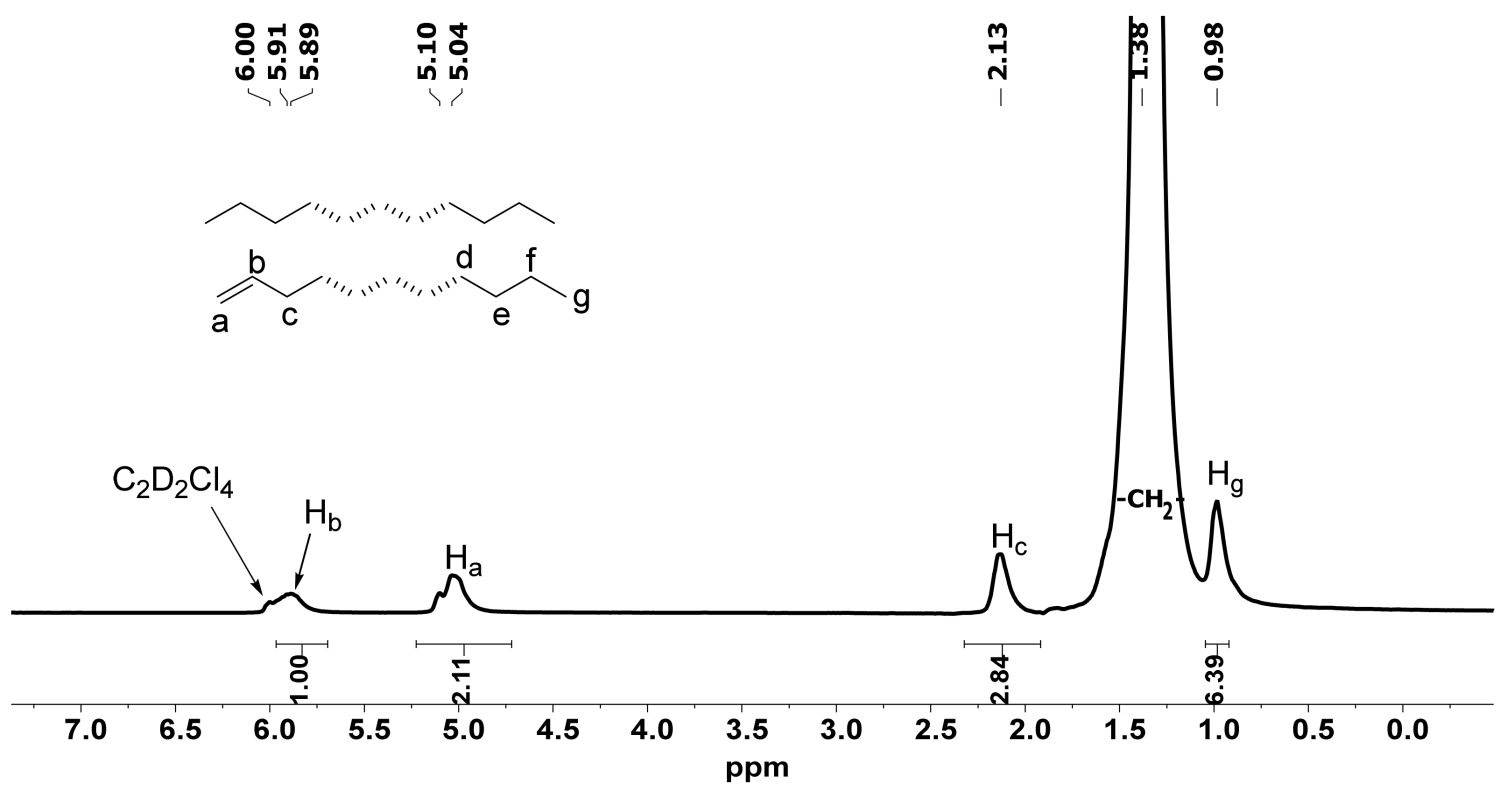
The ^1^H-NMR spectrum of the polyethylene obtained with **Co1**/MMAO (Table 3, entry 9).

**Figure 12:**
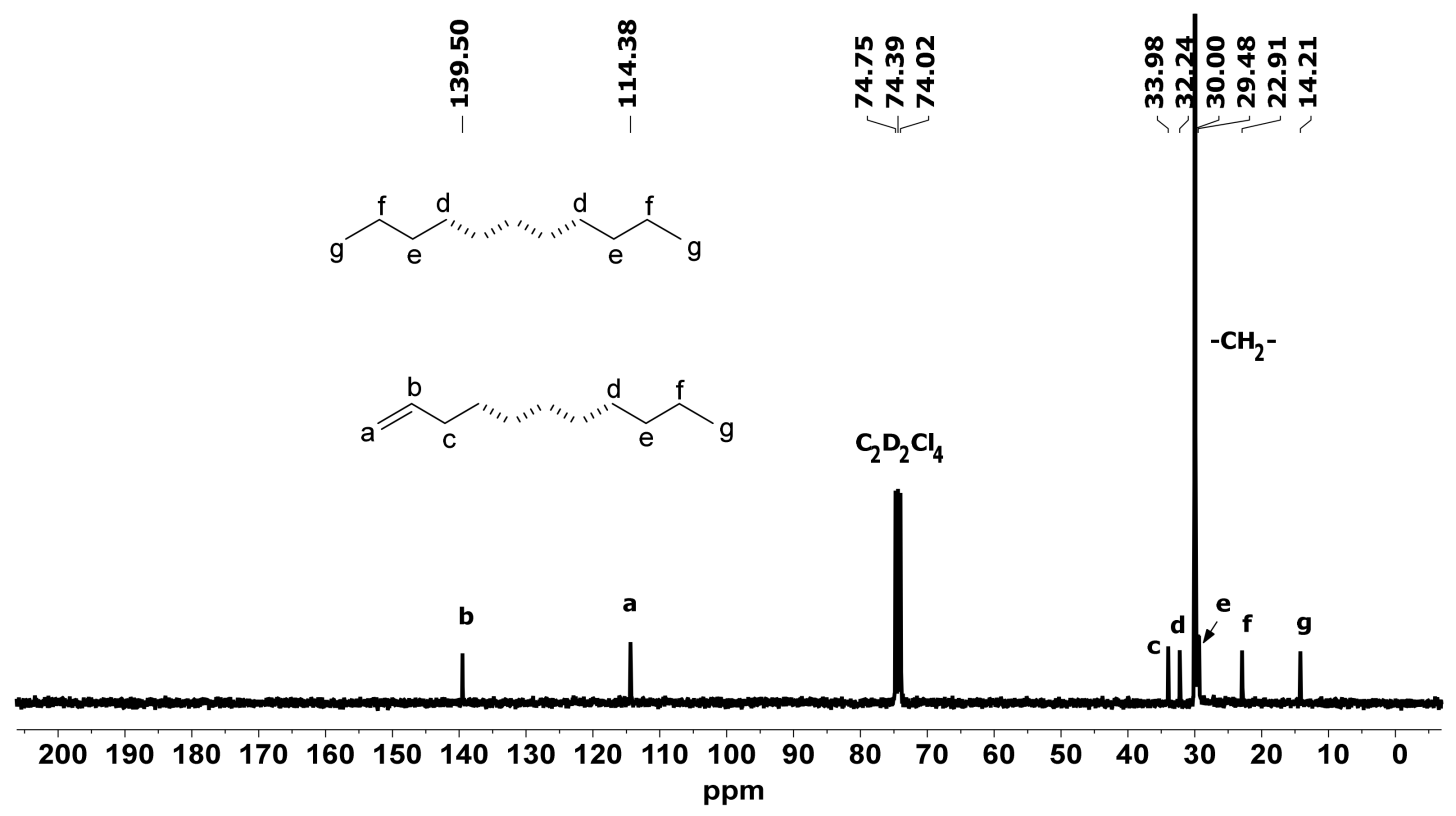
The ^13^C-NMR spectrum of the polyethylene obtained with **Co1**/MMAO (Table 3, entry 9).

A variety of polyethylene samples exhibit high melting temperatures (*T*_m_) from 112.5 °C to 124.5 °C under different conditions, indicating that the microstructure is linear because of the absence of branching content. The detailed examination of product microstructure was completed using high-temperature ^1^H and ^13^C NMR spectroscopy for the **Co1**/MMAO sample obtained at optimum conditions mentioned in entry 9 of Table 3. The existence of PEs with vinyl (CH_2_=CH_2_-) end groups was assured from both ^1^H NMR (H_a_ and H_b_) and ^13^C NMR peaks at C_a_ and C_b_ at δ 139.50 and 114.38 ppm, respectively, in Figure 11 and Figure 12. It is impossible to calculate the exact ratio of saturated to unsaturated PEs (H_a_ to H_g_), due to the peak of the end methyl group overlapping partly with the main backbone (-CH_2_-). However, the ratio of H_a_ to H_g_ is close to 1:3, indicating the presence of saturated PEs components along with high *T*_m_ values of 123.7 °C and 124.5 °C, respectively. A comparatively lower *T*_m_ is related to unsaturated PEs components. Methyl groups at the saturated end of the macromolecules were observed at 14.22 ppm (C_g_) and 32.24, 22.92, and 18.33 ppm (C_d_, C_e_, and C_f_), respectively.

The detailed examination of product microstructure was also completed using high-temperature ^1^H and ^13^C-NMR spectroscopy for the **Co1**/MAO sample obtained at optimum conditions listed in entry 7 of Table 2. The produced polymers have high values of *T*_m_, which range from 121.9 °C to 124.8 °C at different conditions ascribed to the highly linear structures. The signals corresponding to the vinyl (CH_2_=CH_2_-) monomers were observed in both spectra, exhibiting linear polyethylene, as displayed in Figures S3 and S4 of the Supplementary Materials.

Compared with previous reported systems in Chart 1, the catalytic activities of the **Co1**–**Co4** precatalysts in the present study are relatively higher than those of **1B** but lower than other categories. As for the molecular weight of produced polyethylene, it shows that the present cobalt catalytic system has a higher *M*_w_ than **1E** and **1B**, but lower than **1A**, **1C**, and **1D**. Moreover, the current catalytic system exhibits a slightly broader molecular weight distribution (1.84–5.52) relative to the reported analogues, depicting a narrow molecular weight distribution (~2.0). Comparatively, the cobalt complex systems possess relatively good catalytic performance, which has potential application in the practical fields.

### 2.4. Interpretation of Catalytic Activity by MLR Analysis

In the present work, calculations were performed to explore the effect of the substituents on the catalytic activities. The variation of experimental activities for all the cobalt complexes in both cocatalysts, MAO and MMAO, present the same trend, so the results from the MAO runs were selected as representative in order to explore the relationship between the structures of complexes and experimental activities. First, to validate the calculated parameters, the geometries of the complexes **Co1** and **Co4** were optimized and compared to their experimental crystal structures. The comparisons of bond lengths and bond angle between calculated and experimental results are listed in Tables S1 and S2 of the Supplementary Materials. Although the structures at the quartet have higher values of optimized energy (Δ*E*), it is evident that the standardized deviations (*δ*) for bond length and bond angle at the quartet states are smaller than those at the doublet states. Therefore, we chose the calculation results of complexes **Co1**–**Co4** at the quartet state, which is in correspondence with our previous report [38].

**Figure 13:**
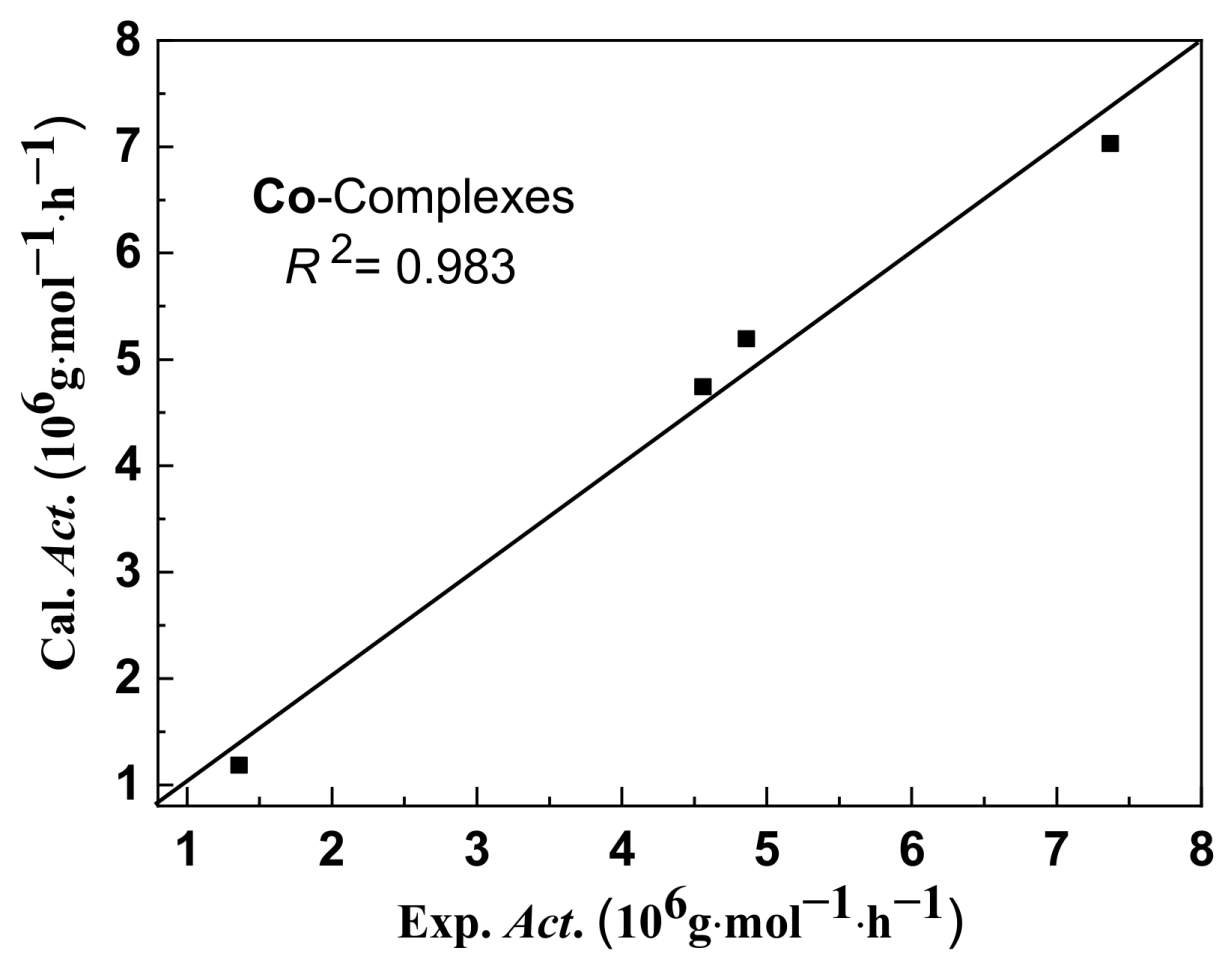
Comparisons between calculated and experimental activities of **Co** complexes by the descriptors of open cone angle (*θ*) and effective net charge (*Q*_eff_).

On the basis of optimized structures, the values of descriptors were calculated and are listed in Table 5. Herein, seven descriptors are considered based on our previous studies [38,39,40], including effective net charge (*Q*_eff_), HOMO–LUMO energy gaps (Δ*ε*_1_, Δ*ε*_2_), energy difference (Δ*E*), Hammett constant (*F*), bite angle (*β*), and open cone angle (*θ*). The detail definition and calculation procedure of each descriptor are provided in Supplementary Materials. For **Co1**–**Co3** complexes in which the *ortho*-substituents *R*^1^/*R*^2^ vary from methyl through ethyl to isopropyl, the values of *Q*_eff_ decrease substantially from 0.527 to 0.493 due to the increase in electron-donating ability of corresponding alkyl groups. Since the ethylene polymerization is electrophilic reaction, the lower net charge on the central metal may cause lower reaction activity. As a result, the experimentally observed activities of these three precatalysts decrease from 7.36 through 4.56 to 1.36 × 10^6^ g (PE) mol^−1^ (Co) h^−1^ (Table 5). Due to the increase in steric hindrance of the alkyl substituents, the value *θ* decreases from 139.83° to 115.98° for **Co1**–**Co3**, along with the decrease of catalytic activities. Furthermore, the energy difference (Δ*E*), HOMO–LUMO energy gap (Δ*ε*_1_), and (Δ*ε*_2_) values present the increasing trend with the decrease of the experimentally determined catalytic activity. There is no discernible correlation between the bite angle (*β*) and the experimental activity. Regarding **Co4**, in which both methyl- and ethyl-substituent occupy the *ortho*-position within the *N*-aryl group, its effective net-charge value is greater than that of **Co2** but lower than that of the **Co1** complex. Meanwhile, due to the increase of hindrance compared to methyl counterpart, its value of *θ* is lower than that of **Co1** and greater than that of **Co2**. In order to calculate the effect of these substituents in terms of the electronic and steric effect on the catalytic activity, a multiple linear regression (*MLR*) analysis is used to explore the relationship between structures with experimental activities [35]. Linear fitting is performed individually for each single descriptor to check its impact on catalytic activities, as shown in Figure S3. Among all the descriptors, it is clear that an open cone angle (*θ*) and effective net charge (*Q*_eff_) exhibit the highest correlations with *R*^2^ values of 0.978 and 0.902, respectively. Therefore, the comparisons between the experimental and the calculated activities of the **Co** complexes using the combination of *θ* and *Q*_eff_ show good correlation, as displayed in Figure 13.

The calculated contribution of each descriptor indicates that the open cone angle (*θ*) is the dominant factor with the contribution value of 62.53%, revealing that the effect of substituents on the catalytic activity is primarily attributable to the steric effect. Higher values of *θ* are relatively favorable to catalytic activity.

## 3. Materials and Methods

### 3.1. General Consideration

All manipulation of water and/or moisture sensitive compounds was performed under a nitrogen atmosphere using a standard high vacuum Schlenk or in a conventional nitrogen-filled glovebox. Before usage, the solvents (toluene and THF) were refluxed for 8–12 h over a suitable drying agent and distilled under a nitrogen environment. Cocatalysts MAO (1.46 M solution in toluene) and MMAO (2.00 M in heptane) were purchased from the Akzo Nobel Corporation. High-purity ethylene was purchased from the Beijing Yansan Petrochemical Company. Other reagents were purchased from Aldrich, Acros, or local suppliers. Crystal data were collected on a MM007HF single-crystal diffractometer with Confocal-monochromatized Mo Kα radiation (λ = 0.71073 Å) at 173(2) K. ^1^H NMR spectra (except for PE) were recorded with a Bruker DMX 400 MHz instrument at room temperature using TMS as an internal standard, while the NMR spectra of the polyethylenes were recorded on a Bruker DMX 300 MHz instrument at 100 °C in deuterated dichlorobenzene (C_6_D_4_Cl_2_) or 1,1,2,2-tetrachloroethane-d_2_ (C_2_D_2_Cl_4_) as a solvent with TMS as an internal standard. A PerkinElmer System 2000 FT-IR spectrometer was used to obtain infrared spectra. A Flash EA 1112 micro-analyzer was used for elemental analysis. The molecular weight (*M*_w_) and molecular weight distribution (*M*_w_/*M*_n_) of polyethylenes were measured on a PL-GPC220 equipment running at 150 °C with 1,2,4-trichlorobenzene as the solvent. The melting temperature (*T*_m_) of polyethylenes was determined using the second scanning run of a PerkinElmer TA-Q2000 DSC analyzer in a nitrogen atmosphere. A sample (4.0–6.0 mg) was heated to 150 °C at a rate of 20 °C/min, held for 5 min at 150 °C to eliminate the thermal history, and then cooled to −20 °C at a rate of 20 °C min^−1^. Before testing the ^13^C NMR spectra of the polyethylenes, they were dissolved to 80 to 100 mg of polyethylene in 1,1,2,2-tetrachloroethane-d_2_ (2 mL) with TMS as an internal standard using a heat gun. The inverse gated ^13^C spectra were then acquired on a Bruker DMX 300 spectrometer at 75.47 MHz in 5 mm standard glass tubes at 100 °C with the number of scans between 1500 and 2000. Used operating conditions: 17.9856 kHz spectral width; 1.8219 s acquisition time; 2.0 s relaxation delay.

### 3.2. General Synthesis of Bulky Anilines ***A1**–**A5***

#### 3.2.1. 2,6-Dimethyl-4-(dibenzopyranyl)aniline (**A1**)

A 250 mL round-bottomed flask was loaded with 2,6-dimethylaniline (1.45 g, 12 mmol) and dibenzopyranol (3 g, 15 mmol). The mixture was stirred for 30 min under nitrogen at 130 °C, and then the catalytic amount of ZnCl_2_ in HCl solution was added dropwise. The reaction mixture was further heated for 2 h, and, upon cooling, the solid was dissolved in DCM (200 mL). It was filtered and washed twice with the saturated solution of NH_4_Cl and NaCl. The organic part was collected, dried over anhydrous Na_2_SO_4_, and concentrated at reduced pressure. The product was recrystallized from DCM using n-heptane to result in a white crystalline solid (1.25 g, 34%). TLC and NMR spectra confirmed that the resultant product is 2,6-dimethyl-4-(dibenzopyranyl)aniline. ^1^H NMR (400 MHz, CDCl_3_, TMS): δ 7.18 (t, *J* = 3.8 Hz, 2H, Ar-H), 7.16 (m, 4H, Ar-H), 7.09 (t, *J* = 7.0 Hz, 2H, Ar-H), 6.76 (s, 2H, Ar-H_m_), 5.08 (s, 1H, –CH–) 2.8 (s, 2H, NH_2_), 2.10 (s, 6H,–CH_3_). ^13^C NMR (100 MHz, CDCl_3_, TMS): δ 151.1, 141.3, 136.3, 129.8, 128.2, 127.5, 125.2, 123.1, 121.9, 116.3, 77.3, 77.0, 76.7, 43.6, 17.7.

#### 3.2.2. 2,6-Diethyl-4-(dibenzopyranyl)aniline (**A2**)

Following the same procedure as for bulky aniline **A1**, the 2,6-diethyl-4-(dibenzopyranyl)aniline was prepared as white solid (1.26 g, 38%). ^1^H NMR (400 MHz, CDCl_3_, TMS): δ 7.15 (t, *J* = 3.8 Hz, 2H, Ar-H), 7.12–7.06 (m, 4H, Ar-H), 6.96 (t, *J* = 3.8 Hz, 2H, Ar-H), 6.76 (s, 2H, Ar-H_m_), 5.12 (s, 1H, –CH–), 3.51 (s, 2H, NH_2_), 2.49–2.43 (m, 4H, –CH_2_–), 1.18 (t, *J* = 7.6 Hz, 6H, –CH_3_). ^13^C NMR (100 MHz, CDCl_3_, TMS): δ 151.1, 140.1, 136.3, 129.7, 128.0, 127.4, 126.2, 125.3, 123.1, 116.3, 77.3, 77.0, 76.7, 43.9, 24.4, 13.0.

#### 3.2.3. 2,6-Diisopropyl-4-(dibenzopyranyl)aniline (**A3**)

In a manner similar to that of bulky aniline **A1**, the 2,6-diisopropyl-4-(dibenzopyranyl)aniline was prepared as a white powder (1.30 g, 36%). ^1^H NMR (400 MHz, CDCl_3_, TMS): δ 7.15 (t, *J* = 6.8 Hz, 2H, Ar-H), 7.10–7.05 (m, 4H, Ar-H), 6.95 (t, *J* = 7.4 Hz, 2H, Ar-H), 6.81 (s, 2H, Ar-H), 5.13 (s, 1H, –CH–), 3.51 (s, 2H, NH_2_), 2.89–2.80 (m, 2H, –CH–), 1.27 (t, *J* = 4.4 Hz, 12H, –CH_3_). ^13^C NMR (100 MHz, CDCl_3_, TMS): δ 151.3, 138.8, 136.0, 132.6, 129.6, 127.4, 125.5, 123.0, 77.3, 77.0, 76.7, 44.3, 28.0, 22.4.

#### 3.2.4. 2-Methyl,6-ethyl-4-(dibenzopyranyl)aniline (**A4**)

Using the same procedure described for bulky aniline **A1**, the 2-methyl,6-ethyl-4-(dibenzopyranyl)aniline was prepared as white powder (1.01 g, 32%). ^1^H NMR (400 MHz, CDCl_3_, TMS): δ 7.11 (t, *J* = 4.2 Hz, 2H, Ar-H), 7.09–6.96 (m, 4H, Ar-H), 6.95 (t, *J* = 4 Hz, 2H, Ar-H), 6.81 (s, 1H, Ar-H_m_), 6.72 (s, 1H, Ar-H_m_), 5.11 (s, 1H, –CH–), 3.51 (s, 2H, NH_2_), 2.51–2.45 (m, 2H, –CH_2_–), 2.09 (s, 3H,–CH_3_), 1.39 (t, *J* = 4Hz, 3H, –CH_3_). ^13^C NMR (100 MHz, CDCl_3_, TMS): δ 151.1, 140.7, 136.3, 129.7, 128.2, 127.6, 127.5 126.3, 125.3, 123.1, 122.4, 116.3, 77.4, 77.0, 76.7, 43.8, 24.4, 17.8, 13.1.

#### 3.2.5. 2,6-Chloro-4-(dibenzopyranyl)aniline (**A5**)

In a manner similar to that of bulky aniline **A1**, the 2,6-chloro-4-(dibenzopyranyl)aniline was prepared as a white powder (1.30 g, 36%). ^1^H NMR (400 MHz, CDCl_3_, TMS): δ 7.15 (t, *J* = 3.8 Hz, 2H, Ar-H), 7.12–7.06 (m, 4H, Ar-H), unifo6.96 (t, *J* = 3.8 Hz, 2H, Ar-H), 6.76 (s, 2H, Ar-H_m_), 5.12 (s, 1H), 3.51 (s, 2H, NH_2_). ^13^C NMR (100 MHz, CDCl_3_, TMS): δ 151.1, 141.7, 135.3, 129.4, 128.1, 127.8, 127.5 126.2, 125.6, 122.3, 119.1, 77.4, 77.4, 76.3, 42.7, 23.3, 22.5.

### 3.3. Synthesis of Cobalt Complexes ***Co1**–**Co5***

#### 3.3.1. Synthesis of 2,6-Bis[1-(4-dibenzopyranyl-2,6-dimethylphenylimino) ethyly]-pyridylcobalt Dichlorides (**Co1**)

The cobalt complex **Co1** was obtained using the one-pot synthesis method. The Schlenk tube was loaded with 2,6 diacetyl pyridine (0.03 g, 0.20 mmol), 2,6-dimethyl-4-(dibenzopyranyl)aniline (0.20 g, 0.60 mmol) and CoCl_2_ (0.02 g, 0.20 mmol) refluxed with acetic acid (30 mL). The reaction continued for 6h at 120 °C, and the volatiles were evaporated to result in a concentrated solution using the pump. The cobalt complex was precipitated using diethyl ether (20 mL), filtered, washed with diethyl ether (3 × 15 mL), and isolated as an air-stable green powder in good yield (0.15 g, 78%). FT-IR (KBr, cm^−1^): 3230 (w), 3001 (w) 1652 (*ν*(C=N), m), 1595 (w), 1577 (w), 1536 (w), 1479 (s), 1450 (s), 1372 (w), 1303 (w), 1301 (w), 1250 (s), 1214 (w), 1148 (w), 1118 (w), 1118 (w), 1096 (w), 1032 (w), 946 (w), 879 (m), 811 (w), 751 (s), 658 (w). Anal. calcd for C_51_H_43_Cl_2_CoN_3_O_2_ (859.76): C, 71.25; H, 5.04; N, 4.89. Found: C, 71.07; H, 5.05; N, 4.85.

#### 3.3.2. Synthesis of 2,6-Bis[1-(4-dibenzopyranyl-2,6-diethylphenylimino) ethyly]-pyridylcobalt Dichlorides (**Co2**)

Following the same procedure described for **Co1**, **Co2** was prepared as a green powder (0.17 g, 94%). FT-IR (KBr, cm^−1^): 2965 (w), 2932 (w), 2875 (w), 1623 (*ν*(C=N), w), 1576 (m), 1543 (w), 1478 (s), 1450 (s), 1371 (w), 1323 (w), 1301 (w), 1251 (w), 1211 (m), 1184 (w), 1150 (w), 1118 (w), 1095 (w), 1030 (w), 981 (w), 940 (w), 881 (m), 808 (w), 749 (s), 676 (w). Anal. calcd for C_55_H_51_Cl_2_CoN_3_O_2_ (915.78): C, 71.13; H, 5.61; N, 4.59. Found: C, 71.92; H, 5.62; N, 4.56.

#### 3.3.3. Synthesis of 2,6-Bis[1-(4-dibenzopyranyl-2,6-diisopropylphenylimino) ethyly]-pyridylcobalt Dichlorides (**Co3**)

Following the same procedure described for **Co1**, **Co3** was prepared as a green powder (0.09 g, 53%). FT-IR (KBr, cm^−1^): 3380 (m), 2961 (m), 2868 (w), 1602(*ν*(C=N), w), 1570 (w), 1550 (w), 1473 (m), 1449 (s), 1369 (w), 1303 (w), 1251 (s), 1211 (w), 1079 (w), 1028 (w), 944 (w), 833 (w), 812 (w), 751 (s), 657 (w). Anal. calcd for C_59_H_59_Cl_2_CoN_3_O_2_ (971.97): C, 72.91; H, 6.12; N, 4.32. Found: C, 73.60; H, 6.60; N, 3.99.

#### 3.3.4. Synthesis of 2,6-Bis[1-(4-dibenzopyranyl-2-methyl, 6-ethylphenylimino) ethyly]-pyridylcobalt Dichlorides (**Co4**)

Following the same procedure described for **Co1**, **Co4** was prepared as a green powder (0.12 g, 63%). FT-IR (KBr, cm^−1^): 3372 (w), 2962 (w), 2284 (w), 2121 (w), 1604 (*ν*(C=N), w), 1543 (m), 1478 (m), 1448 (s), 1373 (w), 1304 (w), 1251 (s), 1210 (w), 1116 (w), 1095 (w), 1029 (w), 945 (w), 880 (w), 812 (w), 753 (s), 670 (w). Anal. calcd for C_53_H_47_Cl_2_CoN_3_O_2_ (887.81): C, 71.70; H, 5.34; N, 4.73. Found: C, 71.97; H, 5.82; N, 4.58.

#### 3.3.5. Synthesis of 2,6-Bis[1-(4-dibenzopyranyl-2,6-chlorophenylimino) ethyly]-pyridylcobalt Dichlorides (**Co5**)

Following the same procedure described for **Co1**, **Co5** was prepared as a green powder (0.30 g, 56%). FT-IR (KBr, cm^−1^): 3376 (w), 2962 (w), 1604 (ν(C=N), w), 1547 (w), 1478 (m), 1448 (s), 1371 (w), 1304 (w), 1251 (s), 1210 (w), 1116 (w), 1095 (w), 1028 (w), 944 (w), 880 (w), 812 (w), 752 (s), 667 (w). Anal. calcd for C_47_H_31_Cl_6_CoN_3_O_2_ (941.42): C, 59.96; H, 3.32; N, 4.46. Found: C, 60.5; H, 3.57; N, 3.94.

### 3.4. X-ray Crystallographic Studies

Two X-ray-quality crystals of complexes **Co1** and **Co4** were grown by the layering of n-hexane into the solution of the corresponding complex in dichloromethane at an ambient temperature. The X-ray studies were performed on an XtaLAB Synergy-R HyPix diffractometer with mirror monochromatic Cu-Kα radiation (λ = 1.54184 Å) at 170.01 or 169.99 K; cell parameters were obtained by global refinement of the position of all collected reflections. The intensities of the Lorentz and polarization effects were corrected, and an empirical absorption was applied. The structures were resolved by the direct methods and refined by full matrix least-squares on *F*^2^. All hydrogen atoms were placed in calculated positions. The structure’s solution and refinement were conducted using SHELXT-97 [41,42].The free solvent molecules were squeezed with PLATON software. The details of the X-ray structure determinations data and structural refinements for **Co1** and **Co4** are provided in Table 6.

### 3.5. Ethylene Polymerization

Ethylene polymerization is conducted in a stainless-steel autoclave (250 mL) equipped with a temperature- and pressure-control system and a mechanical stirrer. The autoclave is initially evacuated and then filled with nitrogen gas. This process is repeated three times after the final evacuation ethylene is introduced. A solution of the corresponding complex (1.5 µmol) in freshly distilled toluene (25 mL) is injected into the autoclave. Another batch of freshly distilled toluene (25 mL) is added, and then the required amount of a cocatalyst (MAO, or MMAO) is injected. After adding another batch of toluene (50 mL), the autoclave is pressurized immediately with ethylene (10 atm), and the content is stirred at a rate of 400 rpm. Upon completion, the stirring is stopped, and the pressure is slowly released. The reaction is quenched with 10% hydrochloric acid in ethanol, and the polymer is washed with ethanol, filtered, and dried under reduced pressure at 40 °C. Finally, the product is weighed. A Schlenk tube is used instead of an autoclave for ethylene polymerization at 1 atm, following a similar procedure.

## 4. Conclusions

The successful incorporation of the 4-dibenzopyranly group at the *para*-position of the *N*-aryl ring of 2,6-bis(imino)pyridyl ligands was performed, and cobalt(II) complexes were synthesized from this ligand framework. The produced cobalt complexes were further characterized by FT-IR, elemental analysis, and X-ray diffraction analysis. The molecular structures of **Co1** and **Co4** depicted the distorted square-pyramidal geometry. Upon activation with MAO or MMAO, good activities were exhibited by these cobalt complex precatalysts up to the value of 7.36 × 10^6^ g (PE) mol^−1^ (Co) h^−1^. The molecular weight and polydispersity indices of resultant polyethylenes are highly dependent on the substitutions of the ligand framework and reaction parameters such as Al/Co molar ratio, reaction temperature, and reaction time. The ^1^H- and ^13^C-NMR spectroscopy depicted the microstructure of the product as the linear polyethylene with the presence of vinyl (CH_2_=CH_2_-) end groups. Moreover, multiple linear regression (*MLR*) analysis was performed to investigate the effect of different substituents, showing the dominant role of the open cone angle and effective net charge on catalytic activity. Meanwhile, the open cone angle presents a relatively higher correlation and bigger contribution with catalytic activity, indicating its dominant role on activity. Compared with previously reported analogues, the present cobalt complexes possess good catalytic performance, such as high catalytic activity and molecular weight, as well slight broader dispersity, showing the potential utility of the dibenzopyranyl group as substituent.

## Figures and Tables

**Table 1:** Selected bond lengths and bond angles for **Co1** and **Co4**.

	Co1	Co4
**Bond Lengths [Å]**		
**Co1–N1**	2.223(3)	2.239 (4)
**Co1–N2**	2.040(3)	2.043(4)
**Co1–N3**	2.225(3)	2.246(4)
**Co1–Cl1**	2.2457(10)	2.2339(13)
**Co1–Cl2**	2.2650(9)	2.2905(14)
**Bond Angles [°]**		
**N1–Co1–N2**	75.04(10)	74.65(15)
**N1–Co1–N3**	148.78(10)	146.73(15)
**N2–Co1–N3**	75.12(10)	74.46(14)
**N1–Co1–Cl2**	97.55(7)	95.57(13)
**N2–Co1–Cl2**	103.83(7)	104.87(13)
**N3–Co1–Cl2**	98.25(8)	104.02(11)
**N1–Co1–Cl1**	98.68(7)	101.17(12)
**N2–Co1–Cl1**	136.03(8)	142.33(13)
**N3–Co1–Cl1**	96.39(8)	95.72(11)
**Cl2–Co1–Cl1**	120.13(5)	112.79(6)

**Table 2:** Ethylene Polymerization by **Co1**/MAO under the conditions *^a^*.

Entry	Precat.	T, °C	t, min	Al/Co	PE, g	Activity *^b^*	*M*_w_^c^	*M*_w_/*M*_n_^c^	*T*_m_, ^o^C *^d^*
**1**	**Co1**	30	30	2000	4.09	5.44	5.02	5.52	123.8
**2**	**Co1**	40	30	2000	4.78	6.38	4.55	5.07	124.8
**3**	**Co1**	50	30	2000	0.62	0.82	3.53	3.70	124.4
**4**	**Co1**	60	30	2000	0.54	0.72	2.29	2.54	124.6
**5**	**Co1**	70	30	2000	-	-	-	-	-
**6**	**Co1**	40	30	2250	5.36	7.14	1.42	1.75	122.4
**7**	**Co1**	40	30	2500	5.52	7.36	2.72	1.85	122.1
**8**	**Co1**	40	30	2750	4.23	5.64	1.63	1.86	123.1
**9**	**Co1**	40	30	3000	3.73	4.97	1.98	2.13	124.4
**10**	**Co1**	40	30	3500	0.19	0.25	1.72	1.84	124.7
**11**	**Co1**	40	5	2500	1.86	14.93	2.42	2.60	124.8
**12**	**Co1**	40	15	2500	3.92	10.45	2.62	3.43	122.9
**13**	**Co1**	40	45	2500	6.38	5.67	2.85	3.56	121.9
**14**	**Co1**	40	60	2500	8.06	5.37	3.12	4.17	122.7
**15 *^e^***	**Co1**	40	30	2500	3.99	5.32	2.04	2.42	121.9
**16 *^f^***	**Co1**	40	30	2500	0.20	0.26	1.89	2.22	124.8

*^a^* General conditions: 1.5 μmol of **Co1**, 100 mL toluene, 10 atm ethylene. *^b^* 10^6^ g of PE mol^−1^(Co) h^−1^. *^c^* 10^3^ g mol^−1^, determined by GPC. *^d^* Determined by DSC. *^e^* 5 atm C_2_H_4_. *^f^* 1 atm C_2_H_4_.

**Table 3:** Ethylene Polymerization by **Co1**/MMAO under the conditions *^a^*.

Entry	Precat.	T, °C	t, min	Al/Co	PE, g	Activity *^b^*	*M*_w_^c^	*M*_w_/*M*_n_^c^	*T*_m_, °C *^d^*
**1**	**Co1**	30	30	2000	0.64	0.86	4.70	4.57	124.5
**2**	**Co1**	40	30	2000	2.73	3.63	3.20	4.32	122.7
**3**	**Co1**	50	30	2000	1.72	2.29	2.17	2.83	123.5
**4**	**Co1**	60	30	2000	1.69	2.26	1.23	1.70	121.9
**5**	**Co1**	70	30	2000	1.65	2.20	1.07	1.72	120.6
**6**	**Co1**	40	30	1000	3.06	4.08	1.23	2.06	122.4
**7**	**Co1**	40	30	1250	3.20	4.25	1.34	1.82	122.5
**8**	**Co1**	40	30	1500	3.46	4.61	1.24	2.34	122.5
**9**	**Co1**	40	30	1750	4.10	5.46	1.17	2.26	121.8
**10**	**Co1**	40	30	2500	0.02	0.02	1.79	2.89	123.2
**11**	**Co1**	40	5	1750	2.11	16.93	1.00	1.89	121.8
**12**	**Co1**	40	15	1750	3.29	8.77	1.09	1.80	121.3
**13**	**Co1**	40	45	1750	4.83	4.29	3.12	1.77	122.3
**14**	**Co1**	40	60	1750	4.99	3.33	4.39	4.79	123.7
**15 *^e^***	**Co1**	40	30	1750	3.10	4.13	1.00	1.90	121.5
**16 *^f^***	**Co1**	40	30	1750	0.95	1.26	0.78	1.64	112.5

*^a^* General conditions: 1.5 μmol of **Co1**, 100 mL toluene, 10 atm ethylene. *^b^* 10^6^ g of PE mol^−1^(Co) h^−1^. *^c^* 10^3^ g mol^−1^, determined by GPC. *^d^* Determined by DSC. *^e^* 5 atm C_2_H_4_. *^f^* 1 atm C_2_H_4_.

**Table 4:** Ethylene Polymerization using **Co1**–**Co5** with either MAO or MMAO as co-catalyst *^a^*.

Entry	Co-Cat.	Precat.	Al/Co	PE, g	Activity *^b^*	*M*_w_^c^	*M*_w_/*M*_n_^c^	*T*_m_, °C *^d^*
**1**	**Co1**	MAO	2500	5.52	7.36	2.72	1.85	122.1
**2**	**Co2**	MAO	2500	3.42	4.56	3.04	3.35	125.7
**3**	**Co3**	MAO	2500	1.02	1.36	85.02	15.22	134.9
**4**	**Co4**	MAO	2500	3.65	4.86	2.95	2.33	124.6
**5**	**Co5**	MAO	2500	-	-	-	-	-
**6**	**Co1**	MMAO	1750	4.10	5.46	1.17	2.26	121.8
**7**	**Co2**	MMAO	1750	2.30	3.06	4.38	3.04	125.4
**8**	**Co3**	MMAO	1750	1.89	2.53	79.85	20.72	131.6
**9**	**Co4**	MMAO	1750	3.40	4.53	3.61	4.28	123.5
**10**	**Co5**	MMAO	1750	-	-	-	-	-

*^a^* General conditions: 1.5 μmol of **Co**, 100 mL toluene, 10 atm ethylene. *^b^* 10^6^ g of PE mol^−1^(Co) h^−1^. *^c^* 10^3^ g mol^−1^, determined by GPC. *^d^* Determined by DSC.

**Table 5:** The values of Hammett constant (*F*), effective net charge (*Q*_eff_), open cone angle (*θ*), bite angle (*β*), energy difference (Δ*E*), and HOMO–LUMO energy gap (Δ*ε*_1,_ Δ*ε*_2_), together with experimental catalytic activities for **Co** complexes.

Complex No.	Descriptors							Activity (10^6^ g·mol^−1^·h^−1^)
	*F*	*Q* [e]	*θ* [°]	*β* [°]	Δ*E* [kcal/mol]	Δ*ε*_1_ [kcal/mol]	Δ*ε*_2_ [kcal/mol]	
**Co1**	0.04	0.527	139.83	147.32	9.72	64.38	88.10	7.37
**Co2**	0	0.507	129.32	141.01	6.61	71.53	88.79	4.56
**Co3**	0.16	0.493	115.98	142.17	11.86	71.59	89.11	1.36
**Co4**	0.02	0.520	132.99	144.12	7.10	70.03	90.86	4.86

**Table 6:** Crystal data and structure refinement for **Co1** and **Co4**.

	Co1	Co4
CCDC No.	2,182,734	2,182,735
Empirical formula	C_51_H_43_Cl_2_CoN_3_O_2_	C_53_H_47_Cl_2_CoN_3_O_2_
Formula weight	859.76	887.81
Temperature/K	170.01 (10)	169.99 (15)
Crystal system	monoclinic	orthorhombic
Space group	P2_1_/n	Pbca
*a/*Å	16.0661 (2)	15.6912 (2)
*b/*Å	16.2364 (2)	19.7010 (2)
*c/*Å	19.4457 (3)	30.6689 (3)
*α/*°	90	90
*β/*°	113.773 (2)	90
*γ/*°	90	90
Volume/Å^3^	4642.11 (13)	9480.75 (18)
*Z*	4	8
*D*_Calcd_ (g/cm^3^)	1.352	1.363
*μ*/mm^−1^	5.359	5.263
*F*(000)	1956.0	4040.0
Crystal size/mm^3^	0.5 × 0.39 × 0.21	2.2 × 1.1 × 0.6
Radiation	CuKα (λ = 1.54184)	CuKα (λ = 1.54184)
2 *θ* range for data collection/°	6.06 to 150.944	5.764 to 151.426
Index ranges	−19 ≤ h ≤ 20, −20 ≤ k ≤ 16, −24 ≤ l ≤ 23	−19 ≤ h ≤ 19, −23 ≤ k ≤ 11, −38 ≤ l ≤ 37
Reflections collected	33,571	74,220
Independent reflections	9252 [R_int_ = 0.0444, R_sigma_ = 0.0372]	9599 [R_int_ = 0.0632, R_sigma_ = 0.0333]
Data/restraints/parameters	9252/0/565	9599/0/583
Goodness-of-fit on *F*^2^	1.021	1.272
Final R indexes [*I* ≥ 2σ (*I*)]	R_1_ = 0.0592, wR_2_ = 0.1473	R_1_ = 0.0950, wR_2_ = 0.2955
Final R indexes [all data]	R_1_ = 0.0760, wR_2_ = 0.1623	R_1_ = 0.1155, wR_2_ = 0.3223
Largest diff. peak/hole/e Å^−3^	0.75/−0.83	0.85/−1.18

## Data Availability

The data presented in this study are available in this article.

## Supplementary Materials

The following supporting information can be downloaded at: https://www.mdpi.com/article/10.3390/molecules27175455/s1, Figure S1: GPC curves of the obtained polyethylene (a); activity and *M*_w_ as a function of ethylene pressure, atm (b) for the **Co1**/MAO system at optimized condition (Table 2, entries 7, 15–16). Figure S2: GPC curves of the obtained polyethylene (a); activity and Mw as a function of ethylene pressure, atm (b) for the **Co1**/MMAO system at optimized condition (Table 5, entries 9, 15–16). Figure S3: The ^1^H-NMR spectrum of the polyethylene obtained with **Co1/**MAO (Table 2, entry 7). Figure S4: The ^13^C-NMR spectrum of the polyethylene obtained with **Co1/**MAO (Table 2, entry 7). Figure S5: Definition of open cone angle (*θ*) of complex. Table S1: Comparisons of bond lengths and bond angles between the calculated geometry and experimental crystal data for complex **Co1** along with the standard deviation (*δ*) and energy variation (Δ*E*) values at various spin states. Table S2: Comparisons of bond lengths and bond angles between the calculated geometry and experimental crystal data for complex **Co4** along with the standard deviation (*δ*) and energy variation (Δ*E*) values at various spin states. Table S3: The values of correlation coefficient (*R*^2^) for **Co** complexes by the combinations of two and single descriptors. Ref. citation of [43,44,45,46,47,48,49,50].

## Sample Availability

Samples of the compounds **Co1**–**Co5** are available from the authors.

## References

[1] Small B.L., Brookhart M., Bennett A.M. (1998). Highly active iron and cobalt catalysts for the polymerization of ethylene. J. Am. Chem. Soc..

[2] Britovsek G.J., Gibson V.C., McTavish S.J., Solan G.A., White A.J., Williams D.J., Kimberley B.S., Maddox P.J. (1998). Novel olefin polymerization catalysts based on iron and cobalt. Chem. Commun..

[3] Johnson L.K., Killian C.M., Brookhart M. (1995). New Pd (II)-and Ni (II)-based catalysts for polymerization of ethylene and α-olefins. J. Am. Chem. Soc..

[4] Killian C.M., Tempel D.J., Johnson L.K., Brookhart M. (1996). Living polymerization of α-olefins using NiII-α-diimine catalysts. Synthesis of new block polymers based on α-olefins. J. Am. Chem. Soc..

[5] Wang Z., Liu Q., Solan G.A., Sun W.-H. (2017). Recent advances in Ni-mediated ethylene chain growth: Nimine-donor ligand effects on catalytic activity, thermal stability and oligo-/polymer structure. Coord. Chem. Rev..

[6] Görl C., Alt H.G. (2007). Influence of the para-substitution in bis (arylimino) pyridine iron complexes on the catalytic oligomerization and polymerization of ethylene. J. Organomet. Chem..

[7] Long Z., Wu B., Yang P., Li G., Liu Y., Yang X.-J. (2009). Synthesis and characterization of para-nitro substituted 2,6-bis (phenylimino) pyridyl Fe (II) and Co (II) complexes and their ethylene polymerization properties. J. Organomet. Chem..

[8] Tomov A.K., Gibson V.C., Britovsek G.J., Long R.J., van Meurs M., Jones D.J., Tellmann K.P., Chirinos J.J. (2009). Distinguishing chain growth mechanisms in metal-catalyzed olefin oligomerization and polymerization systems: C2H4/C2D4 co-oligomerization/polymerization experiments using chromium, iron, and cobalt catalysts. Organometallics.

[9] Yu J., Huang W., Wang L., Redshaw C., Sun W.-H. (2011). 2-[1-(2,6-Dibenzhydryl-4-methylphenylimino) ethyl]-6-[1-(arylimino) ethyl] pyridylcobalt (II) dichlorides: Synthesis, characterization and ethylene polymerization behavior. Dalton Trans..

[10] He F., Zhao W., Cao X.-P., Liang T., Redshaw C., Sun W.-H. (2012). 2-[1-(2, 6-dibenzhydryl-4-chlorophenylimino) ethyl]-6-[1-aryliminoethyl] pyridyl cobalt dichlorides: Synthesis, characterization and ethylene polymerization behavior. J. Organomet. Chem..

[11] Wang S., Li B., Liang T., Redshaw C., Li Y., Sun W.-H. (2013). Synthesis, characterization and catalytic behavior toward ethylene of 2-[1-(4, 6-dimethyl-2-benzhydrylphenylimino) ethyl]-6-[1-(arylimino) ethyl] pyridylmetal (iron or cobalt) chlorides. Dalton Trans..

[12] Lai J., Zhao W., Yang W., Redshaw C., Liang T., Liu Y., Sun W.-H. (2012). 2-[1-(2,4-Dibenzhydryl-6-methylphenylimino)ethyl]-6-[1-(arylimino)ethyl]pyridylcobalt(ii) dichlorides: Synthesis, characterization and ethylene polymerization behavior. Polym. Chem..

[13] Zhang W., Wang S., Du S., Guo C.Y., Hao X., Sun W.H. (2014). 2-(1-(2,4-Bis((di(4-fluorophenyl) methyl)-6-methylphenylimino) ethyl)-6-(1-(arylimino) ethyl) pyridylmetal (iron or cobalt) Complexes: Synthesis, Characterization, and Ethylene Polymerization Behavior. Macromol. Chem. Phys..

[14] Yue E., Zeng Y., Zhang W., Sun Y., Cao X.-P., Sun W.-H. (2016). Highly linear polyethylenes using the 2-(1-(2,4-dibenzhydrylnaphthylimino) ethyl)-6-(1-(arylimino) ethyl)-pyridylcobalt chlorides: Synthesis, characterization and ethylene polymerization. Sci. China Chem..

[15] Mitchell N.E., Anderson W.C., Long B.K. (2017). Mitigating chain-transfer and enhancing the thermal stability of co-based olefin polymerization catalysts through sterically demanding ligands. J. Polym. Sci. Part A Polym. Chem..

[16] Britovsek G.J., Mastroianni S., Solan G.A., Baugh S.P., Redshaw C., Gibson V.C., White A.J., Williams D.J., Elsegood M.R. (2000). Oligomerisation of Ethylene by Bis (imino) pyridyliron and-cobalt Complexes. Chem. Eur. J..

[17] Gibson V.C., Redshaw C., Solan G.A. (2007). Bis (imino) pyridines: Surprisingly reactive ligands and a gateway to new families of catalysts. Chem. Rev..

[18] Xiao L., Gao R., Zhang M., Li Y., Cao X., Sun W.-H. (2009). 2-(1H-2-Benzimidazolyl)-6-(1-(arylimino) ethyl) pyridyl iron (II) and cobalt (II) dichlorides: Syntheses, characterizations, and catalytic behaviors toward ethylene reactivity. Organometallics.

[19] Bianchini C., Giambastiani G., Rios I.G., Mantovani G., Meli A., Segarra A.M. (2006). Ethylene oligomerization, homopolymerization and copolymerization by iron and cobalt catalysts with 2,6-(bis-organylimino) pyridyl ligands. Coord. Chem. Rev..

[20] Appukuttan V.K., Liu Y., Son B.C., Ha C.-S., Suh H., Kim I. (2011). Iron and cobalt complexes of 2,3,7,8-tetrahydroacridine-4,5(1H, 6H)-diimine sterically modulated by substituted aryl rings for the selective oligomerization to polymerization of ethylene. Organometallics.

[21] Smit T.M., Tomov A.K., Britovsek G.J., Gibson V.C., White A.J., Williams D.J. (2012). The effect of imine-carbon substituents in bis (imino) pyridine-based ethylene polymerisation catalysts across the transition series. Catal. Sci. Technol..

[22] Sun W.-H., Kong S., Chai W., Shiono T., Redshaw C., Hu X., Guo C., Hao X. (2012). 2-(1-(Arylimino) ethyl)-8-arylimino-5, 6, 7-trihydroquinolylcobalt dichloride: Synthesis and polyethylene wax formation. Appl. Catal. A Gen..

[23] Du S., Zhang W., Yue E., Huang F., Liang T., Sun W.H. (2016). α, α′-Bis (arylimino)-2,3:5,6-bis (pentamethylene) pyridylcobalt Chlorides: Synthesis, Characterization, and Ethylene Polymerization Behavior. Eur. J. Inorg. Chem..

[24] Huang F., Zhang W., Yue E., Liang T., Hu X., Sun W.-H. (2016). Controlling the molecular weights of polyethylene waxes using the highly active precatalysts of 2-(1-aryliminoethyl)-9-arylimino-5,6,7,8-tetrahydrocycloheptapyridylcobalt chlorides: Synthesis, characterization, and catalytic behavior. Dalton Trans..

[25] Huang F., Zhang W., Sun Y., Hu X., Solan G.A., Sun W.-H. (2016). Thermally stable and highly active cobalt precatalysts for vinyl-polyethylenes with narrow polydispersities: Integrating fused-ring and imino-carbon protection into ligand design. New J. Chem..

[26] Bariashir C., Wang Z., Du S., Solan G.A., Huang C., Liang T., Sun W.H. (2017). Cycloheptyl-fused NNO-ligands as electronically modifiable supports for M (II)(M = Co, Fe) chloride precatalysts; probing performance in ethylene oligo-/polymerization. J. Polym. Sci. Part A Polym. Chem..

[27] Wang Z., Zhang R., Zhang W., Solan G.A., Liu Q., Liang T., Sun W.-H. (2019). Enhancing thermostability of iron ethylene polymerization catalysts through N, N, N-chelation of doubly fused α, α′-bis (arylimino)-2, 3: 5, 6-bis (hexamethylene) pyridines. Catal. Sci. Technol..

[28] Flisak Z., Sun W.-H. (2015). Progression of Diiminopyridines: From Single Application to Catalytic Versatility. Catal. A.

[29] Zada M., Guo L., Ma Y., Zhang W., Flisak Z., Sun Y., Sun W.-H. (2019). Activity and Thermal Stability of Cobalt (II)-Based Olefin Polymerization Catalysts Adorned with Sterically Hindered Dibenzocycloheptyl Groups. Molecules.

[30] Ivanchev S.S., Yakimansky A.V., Rogozin D.G. (2004). Quantum-chemical calculations of the effect of cycloaliphatic groups in α-diimine and bis (imino) pyridine ethylene polymerization precatalysts on their stabilities with respect to deactivation reactions. Polymer.

[31] Pelascini F., Peruch F., Lutz P.J., Wesolek M., Kress J. (2005). Pyridine bis (imino) iron and cobalt complexes for ethylene polymerization: Influence of the aryl imino substituents. Eur. Polym. J..

[32] Liu J.-Y., Zheng Y., Li Y.-G., Pan L., Li Y.-S., Hu N.-H. (2005). Fe (II) and Co (II) pyridinebisimine complexes bearing different substituents on ortho-and para-position of imines: Synthesis, characterization and behavior of ethylene polymerization. J. Organomet. Chem..

[33] Guo L., Zada M., Zhang W., Vignesh A., Zhu D., Ma Y., Liang T., Sun W.-H. (2019). Highly linear polyethylenes tailored with 2, 6-bis [1-(p-dibenzo-cycloheptylarylimino) ethyl] pyridylcobalt dichlorides. Dalton Trans..

[34] Wang Z., Solan G.A., Zhang W., Sun W.-H. (2018). Carbocyclic-fused N,N,N-pincer ligands as ring-strain adjustable supports for iron and cobalt catalysts in ethylene oligo-/polymerization. Coord. Chem. Rev..

[35] Zhang W., Sun W.-H., Redshaw C. (2013). Tailoring iron complexes for ethylene oligomerization and/or polymerization. Dalton Trans..

[36] Britovsek G.J., Bruce M., Gibson V.C., Kimberley B.S., Maddox P.J., Mastroianni S., McTavish S.J., Redshaw C., Solan G.A., Strömberg S. (1999). Iron and cobalt ethylene polymerization catalysts bearing 2, 6-bis (imino) pyridyl ligands: Synthesis, structures, and polymerization studies. J. Am. Chem. Soc..

[37] Wang S., Zhao W., Hao X., Li B., Redshaw C., Li Y., Sun W.-H. (2013). 2-(1-{2,6-Bis[bis(4-fluorophenyl) methyl]-4-methylphenylimino} ethyl)-6-[1-(arylimino) ethyl] pyridylcobalt dichlorides: Synthesis, characterization and ethylene polymerization behavior. J. Organomet. Chem..

[38] Ahmed S., Yang W., Ma Z., Sun W.-H. (2018). Catalytic activities of bis (pentamethylene) pyridyl (Fe/Co) complex analogues in ethylene polymerization by modeling method. J. Phys. Chem. A.

[39] Malik A.A., Yang W., Ma Z., Sun W.-H. (2019). The Catalytic Activities of Carbocyclic Fused Pyridineimine Nickel Complexes Analogues in Ethylene Polymerization by Modeling Study. Catalysts.

[40] Zhang Q., Yang W., Wang Z., Solan G.A., Liang T., Sun W.-H. (2021). Doubly fused N, N, N-iron ethylene polymerization catalysts appended with fluoride substituents; probing catalytic performance via a combined experimental and MLR study. Catal. Sci. Technol..

[41] Kratzert D., Holstein J.J., Krossing I. (2015). DSR: Enhanced modelling and refinement of disordered structures with SHELXL. J. Appl. Crystallogr..

[42] Sheldrick G.M. (2015). SHELXT–Integrated space-group and crystal-structure determination. Acta Crystallogr. Sect. A Found. Adv..

[43] Hansch C., Leo A., Taft R. (1991). A survey of Hammett substituent constants and resonance and field parameters. Chem. Rev..

[44] Le T., Epa V.C., Burden F.R., Winkler D.A. (2012). Quantitative structure–property relationship modeling of diverse materials properties. Chem. Rev..

[45] Delley B. (1990). An all-electron numerical method for solving the local density functional for polyatomic molecules. J. Chem. Phys..

[46] Delley B. (2000). From molecules to solids with the DMol 3 approach. J. Chem. Phys..

[47] Becke A.D. (1988). A multicenter numerical integration scheme for polyatomic molecules. J. Chem. Phys..

[48] Dolg M., Wedig U., Stoll H., Preuss H. (1987). Energy-adjusted abinitio pseudopotentials for the first row transition elements. J. Chem. Phys..

[49] Bergner A., Dolg M., Küchle W., Stoll H., Preub H. (1993). Ab initio energy-adjusted pseudopotentials for elements of groups 13–17. Mol. Phys..

[50] Li Z., Bian K., Zhou M. (1997). Excel for Windows95 Encyclopaedia.

