# Supplementary material for: Catalytic Performance of Cobalt(II) Polyethylene Catalysts with Sterically Hindered Dibenzopyranyl Substituents Studied by Experimental and MLR Methods
Source: Molecules. 2022 Aug 25;27(17):5455. doi: 10.3390/molecules27175455 (PMC9458042; doi:10.3390/molecules27175455)
Supplement: Supplementary file 1 [file molecules-27-05455-s001.zip › molecules-1865072-supplementary.pdf]

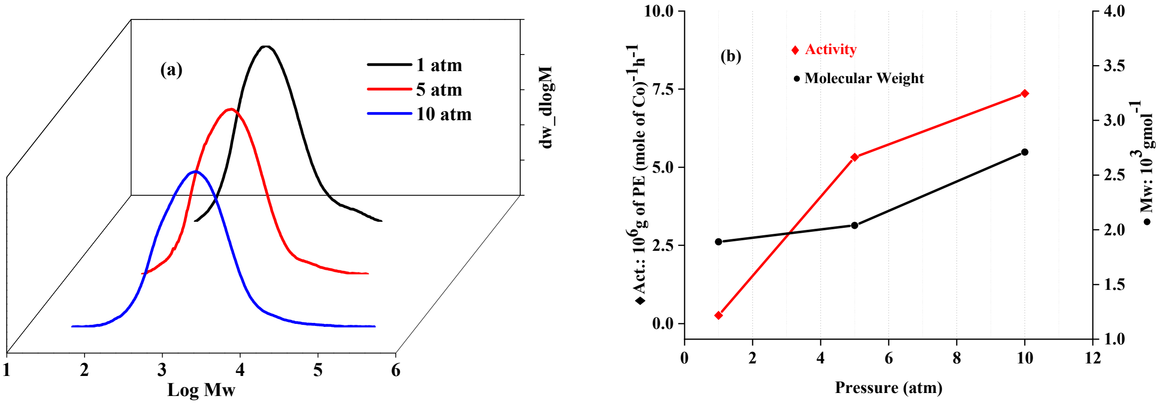

Figure S1. GPC curves of the obtained polyethylene (a); activity and *M*w as a function of ethylene pressure, atm (b) for the **Co1**/MAO system at optimized condition (Table 2, entries 7, 15–16)

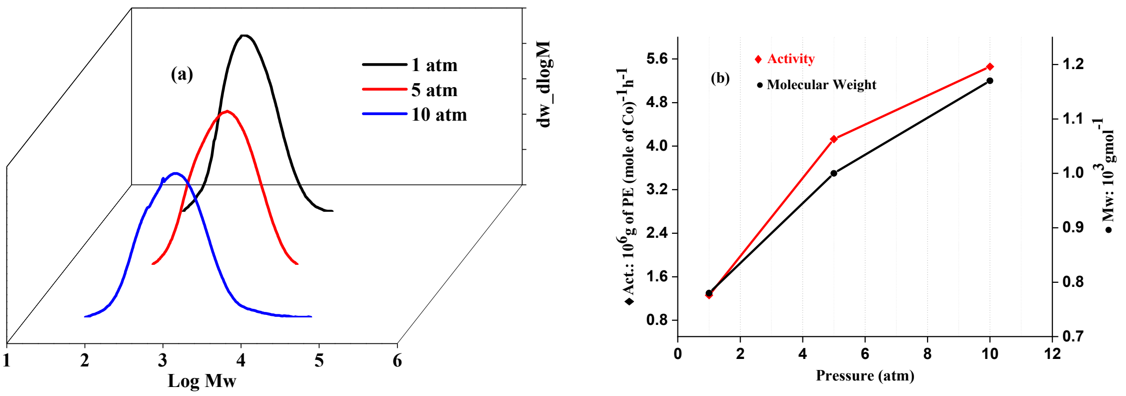

Figure S2. GPC curves of the obtained polyethylene (a); activity and Mw as a function of ethylene pressure, atm (b) for the **Co1**/MAO system at optimized condition (Table 5, entries 9, 15–16)

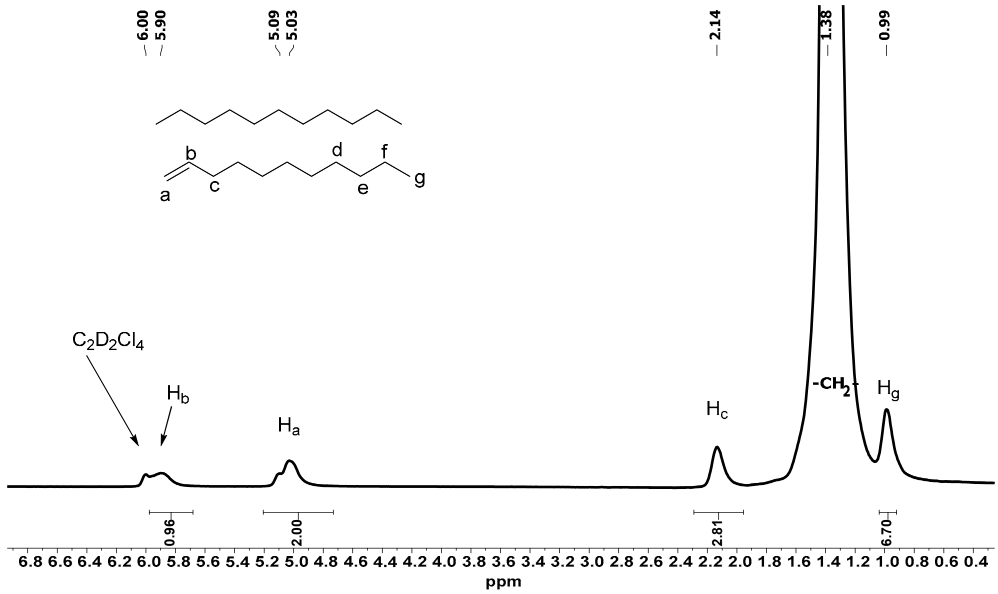

Figure S3. The ^{1}H-NMR spectrum of the polyethylene obtained with **Co1/MAO** (Table 2, entry 7).

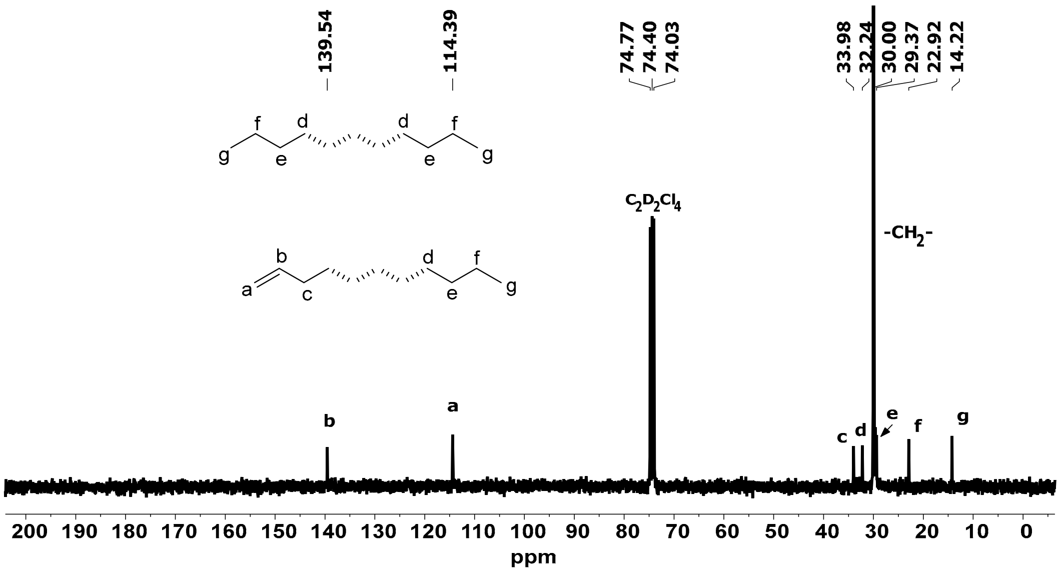

Figure S4. The ^{13}C-NMR spectrum of the polyethylene obtained with **Co1/MAO** (Table 2, entry 7).

Geometry Optimization and Descriptors Calculation

All the calculations are performed by density function theory (DFT) in Dmol3 program in order to optimize the geometry of molecular structures.[1, 2]. The electronic structures of the molecular systems are optimized by the generalized gradient approximation (GGA) [3] and Becke-Perdew (BP) [4] exchange correlation functional combined with the double numerical basis sets with polarization functions (DNP) [1] using effective core potentials [5, 6]. For geometry optimization, the convergence criteria of energy, maximum force and displacement are 2.0×10^{-5} Hartree, 4.0×10^{-3} Hartree per Bohr and 5.0×10^{-3} Å, respectively. The convergence criterion for self-consistent field (SCF) calculation is 1.0×10^{-5} Hartree.

Table S1. Comparisons of bond lengths and bond angles between the calculated geometry and experimental crystal data for complex **Co1** along with the standard deviation (δ) and energy variation (ΔE) values at various spin states.

Co1	Exp.	Doublet	Quartet
	**Bond Lengths [Å]**		
**Co1–N1**	2.223(3)	2.007	2.361
**Co1–N2**	2.040(3)	1.845	2.066
**Co1–N3**	2.225(3)	2.007	2.229
**Co1–Cl1**	2.2457(10)	2.205	2.222
**Co1–Cl2**	2.2650(9)	2.309	2.271
δ		5.51	2.82
	**Bond Angles [°]**		
**N1–Co1–N2**	75.04(10)	80.66	72.65
**N1–Co1–N3**	148.78(10)	158.65	147.32
**N2–Co1–N3**	75.12(10)	80.75	76.15
**N1–Co1–Cl2**	97.55(7)	95.27	94.43
**N2–Co1–Cl2**	103.83(7)	90.95	97.57
**N3–Co1–Cl2**	98.25(8)	95.46	99.08
**N1–Co1–Cl1**	98.68(7)	97.28	94.06
**N2–Co1–Cl1**	136.03(8)	162.57	137.77
**N3–Co1–Cl1**	96.39(8)	97.29	102.67
**Cl2–Co1–Cl1**	120.13(5)	106.47	123.77
δ		9.56	3.86
ΔE(kcal/mol)		0	9.72

Table S2. Comparisons of bond lengths and bond angles between the calculated geometry and experimental crystal data for complex **Co4** along with the standard deviation (δ) and energy variation (ΔE) values at various spin states.

Co4	Exp.	Doublet	Quartet
	**Bond Lengths [Å]**		
**Co1–N1**	2.239 (4)	2.026	2.340
**Co1–N2**	2.043(4)	1.846	2.067
**Co1–N3**	2.246(4)	2.001	2.236
**Co1–Cl1**	2.2339(13)	2.210	2.235
**Co1–Cl2**	2.2905(14)	2.307	2.271
δ		5.46	2.16
	**Bond Angles [°]**		
**N1–Co1–N2**	74.65(15)	80.59	73.01
**N1–Co1–N3**	146.73(15)	159.44	144.12
**N2–Co1–N3**	74.46(14)	80.868	74.72
**N1–Co1–Cl2**	95.57(13)	94.495	98.42
**N2–Co1–Cl2**	104.87(13)	90.501	93.77
**N3–Co1–Cl2**	104.02(11)	94.48	98.94
**N1–Co1–Cl1**	101.17(12)	97.41	98.19
**N2–Co1–Cl1**	142.33(13)	158.02	144.79
**N3–Co1–Cl1**	95.72(11)	96.44	99.13
**Cl2–Co1–Cl1**	112.79(6)	111.48	121.41
δ		8.27	5.27
ΔE(kcal/mol)			7.10

After the optimization of the complex, we calculate the seven descriptors of electronic and steric effects. Effective net charge (Q_{eff}) is calculated by using equation S1 which defines the difference of charge on central metal (Q_{CM}) and variation between two halogen atoms ($\Delta Q_{halogens}$). The HOMO–LUMO energy gaps ($\Delta\varepsilon_1$, $\Delta\varepsilon_2$) are actually the energy gap between complex's LUMO/HOMO (E_{LC}/E_{HC}) and ethylene's HOMO/LUMO orbitals (E_{HE}/E_{LE}) as calculated by equation S2 and S3, respectively.

$$Q_{eff} = Q_{CM} - \Delta Q_{halogens} \quad \text{S1}$$

$$\Delta\varepsilon_1 = E_{LC} - E_{HE} \quad \text{S2}$$

$$\Delta\varepsilon_2 = E_{LE} - E_{HC} \quad \text{S3}$$

where in equation S2, E_{LC} is LUMO orbital energy of complex and E_{HE} is HOMO orbital energy of ethylene, for equation S3, it is vice versa.

Energy difference (ΔE) can be explained in terms of different optimized energy of **Ni** complex between singlet and triplet states. The values of Hammett constant (F) are taken from literature [7] and depends on the type of substituents. Steric effect is calculated on the basis of optimized structure of complex. The bite angle (β) are measured as the angle of **N1–Ni–N2** in complex. The open cone angle (θ) are obtained according to equation S4 accounting for the space to accommodate the incoming monomer.

$$\theta = 360^{o} - \left[\angle C_1 + arcsin\frac{r_2}{L_2} + \angle D_1 + arcsin\frac{r_1}{L_1}\right] \qquad \text{S4}$$

Optimized geometry structure of precatalyst is very important to calculate $\angle C_1$, $\angle D_1$, and L_1, L_2. r_1 and r_2 are the van der Waals radius of the outmost atom as shown in (Figure S5).

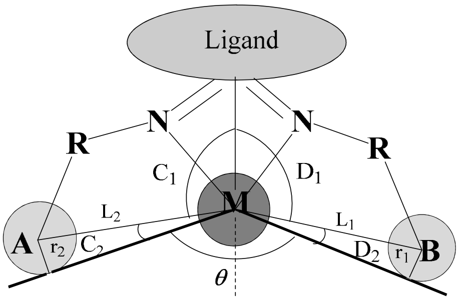

Figure S5. Definition of open cone angle (θ) of complex.

To exhibit the association of structural descriptors with experimental activities, we use the following multiple linear regression analysis (*MLRA*) as in equation S5:

$$Act.(10^{a}\ \mathrm{g \cdot mol^{-1} \cdot h^{-1}}) = \sum_{i=1}^{N} m_o + m_i X_i \qquad \text{S5}$$

where N is the number of structural descriptors to establish the correlation with catalytic activities. In this study, we consider to choose two different descriptors from the seven descriptors, so the value of N is 2. X_i is the value of each descriptor. The regression coefficients m_o and m_i are obtained after linear fitting using the LINEST function in Microsoft Excel.[8]

To analyze the contribution of each structural descriptor to the catalytic activity of the complex, the values of descriptors and catalytic activities are standardized by Z-score method.[9, 10] Contribution values can be calculated for each descriptor by using the standardized values as shown in the equation S6:

$$Contribution\,\% = \frac{\sum_{j=1}^{M}\frac{|\bar{w}_i \cdot \bar{X}_{ij}|}{\sum_{i=1}^{N}|\bar{w}_i \cdot \bar{X}_{ij}|}}{M} \times 100\% \qquad \text{S6}$$

where, M is corresponding to the number of complexes for one series and N describes the number of structural descriptors (2). $\bar{w}$ and $\bar{X}$ are the standardized values of linear fitting coefficients and one concerned descriptor, respectively.

Table S3. The values of correlation coefficient (R^2) for **Co** complexes by the combinations of two and single descriptors.

**Two Descriptors**	R^2	**Two Descriptors**	R^2	**Single descriptor**	R^2
F, $\Delta\varepsilon_1$	0.999	Q, $\Delta\varepsilon_1$	0.916	θ	0.978
ΔE, θ	0.986	F, Q	0.914	Q	0.902
$\Delta\varepsilon_1$, θ	0.987	Q, ΔE	0.903	$\Delta\varepsilon_1$	0.687
ΔE, $\Delta\varepsilon_1$	0.995	Q, β	0.903	F	0.534
F, θ	0.982	$\Delta\varepsilon_2$, θ	0.999	β	0.533
Q, $\Delta\varepsilon_2$	0.977	ΔE, β	0.888	ΔE	0.182
F, ΔE	0.969	$\Delta\varepsilon_1$, β	0.714	$\Delta\varepsilon_2$	0.070
θ, β	0.980	$\Delta\varepsilon_1$, $\Delta\varepsilon_2$	0.704		
F, β	0.957	F, $\Delta\varepsilon_2$	0.657		
Q, θ	0.983	$\Delta\varepsilon_2$, β	0.566		
ΔE, $\Delta\varepsilon_2$	0.392				

It is clear that there are also other combinations of two descriptors showing high correlations with R^2 over 0.95. Actually, the combination of open cone angle and effective net charge was

only considered, mainly based on the correlation results between each single descriptor and catalytic activity. These two descriptors have the highest correlations, presenting more important role on activity.

References

1- Hansch, C.; Leo, A.; Taft, R. A survey of Hammett substituent constants and resonance and field parameters. *Chemical reviews* **1991**, *91*, 165-195.
2- Le, T.; Epa, V. C.; Burden, F. R.; Winkler, D. A. Quantitative structure–property relationship modeling of diverse materials properties. *Chemical reviews* **2012**, *112*, 2889-2919.
3- Delley, B. An all-electron numerical method for solving the local density functional for polyatomic molecules. *The Journal of chemical physics* **1990**, *92*, 508-517.
4- Delley, B. From molecules to solids with the DMol 3 approach. *The Journal of chemical physics* **2000**, *113*, 7756-7764.
5- Becke, A. D. A multicenter numerical integration scheme for polyatomic molecules. *The Journal of chemical physics* **1988**, *88*, 2547-2553.
6- Dolg, M.; Wedig, U.; Stoll, H.; Preuss, H. Energy-adjusted abinitio pseudopotentials for the first row transition elements. *The Journal of chemical physics* **1987**, *86*, 866-872.
7- Bergner, A.; Dolg, M.; Küchle, W.; Stoll, H.; Preub, H. Ab initio energy-adjusted pseudopotentials for elements of groups 13–17. *Molecular Physics* **1993**, *80*, 1431-1441.
8- Li, Z.; Bian, K.; Zhou, M.: Excel for Windows95 Encyclopaedia. Electronics Industry Press, Beijing, 1997.
